# Psychometric analysis of the Oral Health Behavior Social Support Short Form scales (OHBSS-SF) among Mexican-American adults

**DOI:** 10.21203/rs.3.rs-10236791/v1

**Published:** 2026-07-20

**Authors:** Tracy L. Finlayson, Cristian Garcia-Alcaraz, Vanessa L. Malcarne, Guadalupe X. Ayala, Kristin S. Hoeft, Mark Ryder, Stuart A. Gansky, Lourdes S. Martinez, Melody K. Schiaffino, Erin Dougherty, Nannette Stamm, Gerardo Maupomé

**Affiliations:** 1 School of Public Health, San Diego State University, San Diego, California, USA; 2 Institute for Behavioral and Community Health, San Diego, California, USA; 3 San Diego State University/University of California San Diego Joint Doctoral Program in Clinical Psychology; 4 Department of Psychology, San Diego State University, San Diego, California, USA; 5 School of Dentistry, University of California San Francisco, San Francisco, California, USA (majority of work done at UCSF); 6 Department of Bioengineering, School of Medicine, Stanford University, Stanford, California, USA; 7 School of Communication, San Diego State University, San Diego, California, USA; 8 University of California San Diego School of Medicine (majority of work done while affiliated with 1 & 2), La Jolla, California, USA; 9 El Rio Health, Tuscon, Arizona, USA; 10 Vista Community Clinic, Vista, California, USA; 11 Richard M. Fairbanks School of Public Health, Indiana University, Indianapolis, Indiana, USA

**Keywords:** Psychometric properties, scale validation, short scale, social support, oral health, brushing, flossing, oral hygiene, dental care utilization, Mexican-American

## Abstract

**Background:**

Social support is an important psychosocial resource that can facilitate engaging in healthy behaviors, including oral health behaviors. Nine scales assessing Oral Health Behavior Social Support (OHBSS) for brushing, flossing and seeking dental care were developed and validated in the English and Spanish languages. The full-length scales included 37 required items to assess social support from three sources: family, health providers, and others/friends. The OHBSS-SF scales cover three oral health behaviors, use six items each to assess social support for brushing, flossing and dental care, and retain the nine-scale structure. Response options range from one (never) to four (always), with higher scores reflecting more frequent receipt of social support. This paper describes the item reduction process/outcomes and presents model fit indices and psychometric properties of the 17-item OHBSS-Short Form (OHBSS-SF) scales.

**Methods:**

A large Mexican-American young adult sample completed an online survey in English or Spanish (N=502). Participants completed the OHBSS-SF along with several other validated scales assessing general social support, dental fear, and acculturation. The survey also queried oral health behaviors of interest and socio-demographic characteristics. A subset of participants (n=56) also completed the OHBSS-SF items a second time two to six weeks later to assess test-retest reliability. Model fit indices and psychometric properties for each of the nine OHBSS-SF scales were calculated for the full sample and subgroups stratified by the three study design characteristics of language (English/Spanish), sex (male/female), and marital status (single/married).

**Results:**

The OHBSS-SF scales correlated very highly with the full-length versions (r=0.95–0.98), and all OHBSS-SF scales exhibited acceptable model fit indices. The OHBSS-SF demonstrated stability in the full sample and in Spanish, with good to excellent intra-class correlations, supporting test-retest reliability. Patterns of moderate correlations supported convergent validity with validated general social support measures. Patterns of weak correlations suggested divergent validity between OHBSS-SF and other non-social support measures.

**Conclusions:**

The OHBSS-SF demonstrated acceptable psychometric properties. A few significant differences by subgroup characteristics were identified. The OHBSS-SF scales reduce respondent burden and are a useful tool to ascertain frequency of receipt of oral health behavior social support from family, health providers, and other/friend sources.

## INTRODUCTION

### Oral health behaviors and Mexican-American young adults

In the United States (US), Mexican-American children and adults experience a higher burden of oral diseases than non-Hispanic Whites, based on National Health and Nutrition Examination Survey (NHANES) 2017-March 2020 data (1). Among all US adults (age 20–64), men had significantly more untreated decay than women (1). In the 2017–2018 NHANES data, men also exhibited poorer oral health behaviors than women (2). Among Mexican-origin men and women, women reported more frequent engagement in daily hygiene (3). Relevant oral health behaviors include daily hygiene (brushing teeth and flossing or cleaning in between teeth, especially after eating), and timely visits to dental health professionals for cleanings, exams, and needed treatment. A consistent practice of oral hygiene is desirable since it can reduce the risk of oral diseases (4, 5).

### Social Support

Social support can facilitate engaging in health-promoting behaviors (e.g., physical activity, eating a healthy diet), and conversely, is associated with engaging in less risky health behaviors (e.g., alcohol consumption)(6, 7). A recent systematic review and meta-analysis concluded that perceived social support had significant positive association with physical health and significant negative association with engagement in risky behaviors (8). The review also explored the role of different types and sources of social support, and found some moderating effects on the relationships between social support and physical health and risk-taking. A robust literature shows that perceived available social support is also important for positive health outcomes (9–11) and serves as a potential resource to mitigate stress (12).

Social support is a multi-dimensional construct, with no single gold standard measure (13). Various validated general measures operationalize social support depending on the underlying theoretical perspective, dimensions of interest (e.g., source and type), and behaviors under study (14). In addition, there are both structural and functional components of social support. Structural components of social support include the number of social ties with others and frequency of contact with others; these are more typically studied in terms of social networks and their characteristics (15). Perceived availability of functional components are typically measured when assessing social support (16, 17), and include measures of reported frequency of social support and/or satisfaction with received emotional, informational, appraisal and instrumental support (14, 18). In a study of Mexican immigrants living in the midwestern US, social network characteristics were related to dental care utilization; specifically network size, network members’ own utilization, and extent to which dental care was discussed were positively associated with dental visits (19).

Social support operates differently across diverse groups of people, including groups based on gender, stage of life, and marital status. For example, sources and types of social support have been shown to differ between men and women, warranting closer examination and comparison (20). US-based studies suggest women tend to give and take more emotional support than men, and have different gender-based social roles and behavioral expectations (21–23). Also in nearly all states in the US, legal adulthood begins at age 18. The transition to young adulthood is often marked by significant increases in independence, potentially moving away from family, and changes in interpersonal relationships and social network composition (24, 25). In a meta-analysis, social network size was found to be stable within family networks during adolescence through young adulthood, but expanded by about one-quarter with growth in new non-family ties (26).

Primary sources of social support may shift from family to friends and romantic partners/significant others as the people with which a person most frequently interacts. However, this may not always be the case among Mexican-American families. As represented in the concept of *familismo* (27, 28), many Mexican-Americans maintain close family ties and obligations to their family across their lifespan (29). The role of *familismo* in the context of oral health was apparent in a study of Mexican-American adults who identified that they discussed oral health and dental problems with family members (30). Social support and social network characteristics have been studied in the Hispanic Community Health Study/Study of Latinos (HCHS/SOL); among recent immigrants, structural social support was protective against dental caries (cavities) (31) and tooth loss (32).

Proximity to and amount of time spent with neighbors, peers, and co-workers may also increase during this stage of life (22). Marital status may also influence an adult’s social network and strongly shape the perceived sources and types of social support readily available (33). Marriage is a normative life event during young adulthood (26). Marriage typically expands family networks, and married couples generally give and receive more social support from their partners (34). When assessing the independent effects of marital status and gender, marital status and not gender accounted for more differences in perceived social support (33). While other factors and life events (e.g., parenthood, widowhood, changing jobs) can also shift social networks and available social support, differences in perceived social support by marital status should be considered.

Behavior-specific measures of social support have been useful in understanding how social supports affects the health and health behaviors of Mexican-Americans. These include measures of social support for healthy eating and physical activity choices (35). Social support has been targeted successfully in health promotion interventions with Mexican-origin families (e.g., dietary behaviors (36–38), physical activity (39), and chronic disease management (40, 41). In a sample of Latinx adults, larger network size was associated with meeting physical activity goals (42). In the context of oral health behaviors specifically, a recent systematic review suggested that social support is promising for immigrants and ethnic minorities, including Mexican-Americans, in promoting oral health behaviors and positively associated with oral health outcomes (43).

### Developing Oral Health Behavior Social Support (OHBSS) Scales

Developing a social support measure for research that is context-specific to oral health behaviors for a target population group (in this case, Mexican-Americans) is essential in understanding the relationship between social support and oral health behaviors (44). Among Mexican-American young adults, nine new OHBSS scales were created and validated in two languages simultaneously (English and Spanish); the full-length OHBSS scales includes 37 required items, plus an optional set of up to seven additional items (45). These scales queried social support for three different oral health behaviors (brushing, flossing, and accessing dental care), from three different sources (family, healthcare providers, others/friends), and included items capturing multiple types of social support (informational, instrumental, emotional, and appraisal). Nine total scales were developed for each different behavior-source combination. The full length OHBSS scales skew towards informational social support overall, comprising about half the items in each scale.

The rigorous process of scale creation, item bank development and item refinement, and assessment of structural validity of these full-length scales in both English and Spanish are reported elsewhere (45). The psychometric properties of the full-length OHBSS scales overall and in both English and Spanish were adequate (46). Additionally, a social network analysis was employed to characterize convergent and divergent validity using the full length OHBSS scales (47).

The full-length OHBSS scales included 12 brushing social support items, 11 flossing social support items, and 14 items for dental care social support. One general oral hygiene item in the brushing set is also scored with the flossing set of items for 12 items total. The self-administered near-final full-length OHBSS scales could be completed relatively quickly (17–22 minutes). Scale length varied from 39 required items up to 48 items, depending on how many of the optional questions applied to the respondent (45). In the final full-length OHBSS scales, a few required and optional items were removed, yielding 37 required items and up to seven optional items. Cognitive effort and response burden was considered when developing the OHBSS scales, and a matrix response format was used to ask the same item for each of the three potential sources of support in order to minimize item repetition.

Having the full-length OHBSS scales encompass three oral health behaviors, comprehensively assess four types of social support, from three different sources of social support groups is beneficial. However, the full-length scales may be considered long, and overall response burden may be considered high. A recent systemic review and meta-analysis found that response rates were significantly associated with survey length (response rates were lower for longer instruments), though it is unclear how the survey content may also play a role in response burden (48). Response burden may be affected by other aspects of the instrument besides length, including the visual layout, item ordering, and clarity of instructions and items (49, 50). These findings warrant the exploration of a short version of the OHBSS scales.

The present report is focused on young adult Mexican-American men and women in the southwestern US, characterizing social support for oral health behaviors, now describing shortened versions of the full-length OHBSS scales. Such description encompasses the creation and validation of the 17-item OHBSS Short-Form (OHBSS-SF) scales. The process of selecting items retained in the OHBSS-SF is described in the present report, along with evidence for the following psychometric properties: internal consistency; structural, convergent, divergent and predictive validity; and test-retest reliability. These are summarized for the full sample as well as by study design balance characteristic subgroup: language preference (English vs. Spanish), sex (male vs. female), and marital status (single vs. married/partnered, hereafter presented as “married”).

## METHODS

### Study Design

Online survey data collection occurred April 2022 to February 2023, from a large diverse sample of young adults of Mexican heritage (full sample, N =502). Additional data were collected from two convenience subsamples following survey completion. Subsample 1 involved dental exams at two participating clinic sites, and only participants living near one of these two California sites were invited to schedule a study dental exam appointment (not discussed further). All participants were invited to opt-in to Subsample 2 and complete a repeat survey with OHBSS scales two to six weeks after completing their first survey.

An important component of the study design was to recruit and enroll a cross-classified sample of participants balanced by language preference, sex, and marital status in the full sample. These three characteristics were monitored throughout recruitment and enrollment to support meaningful comparisons across these characteristics. Balance by language preference was prioritized over balance by sex or marital status, given the necessity to test for meaningful equivalence in the new scales in both English and Spanish. Balancing sex and marital status across study participants was important, given potential differences in social support.

The San Diego State University (SDSU) Institutional Review Board (IRB) and a clinical study oversight committee appointed by the National Institutes of Dental and Craniofacial Research (NIDCR) reviewed and approved protocols before data collection.

### Setting and Participants

Participant eligibility and recruitment efforts have been detailed elsewhere (45). Briefly, eligibility criteria included: self-identifying as Mexican-origin, being a young adult between the ages of 21–40 years, residing in one of eight counties: San Diego, Imperial, Riverside, or Orange county in California (CA); or Pima, Cochise, Santa Cruz, or Yuma county in Arizona (AZ). Participants had to select their preferred language, and be able to read, write and speak in either English or Spanish. A temporary exclusion criterion applied to women who were pregnant, and they were offered the option to be waitlisted to participate later. Other exclusion criteria included: edentulism (lacking all natural teeth), not being able to provide informed consent, or needing pre-medication before dental procedures. Potential participants could self-screen online, then trained study staff confirmed eligibility, obtained written informed consent in English or Spanish and sent them a unique survey link. Surveys took approximately one hour to complete online. Separate informed consent forms were obtained before participation in either subsample.

### Item selection for the Oral Health Behavior Social Support Short-Form (OHBSS-SF)

The items included in the OHBSS-SF were selected from the validated set of 37 items included in the required full-length version of the scales (46). The goal was to reduce the length without adversely affecting the validity and reliability of the scales. During the final stages of item selection for the full-length OHBSS scales, the expert panel reviewed the response patterns and ensured that parallel brushing and flossing items were intentionally retained when considering alternately worded similar items (45). The panel recognized that brushing and flossing were often performed together daily. The parallel set of hygiene items appeared to be a reasonable start to a shorter subset of items. A subset of the expert panel met and closely reviewed the required 37 items in the OHBSS scales to identify dental care items that appeared to align with the parallel brushing and flossing items. The identified smaller set of items captured informational and instrumental types of social support, the two dominant types of support in the full-length OHBSS scales. Table 1 presents the items selected for the OHBSS-SF. All the OHBSS-SF items have been labeled a-f, and a crosswalk with the original item numbers is presented in Table 1. The Dental Care OHBSS-SF scale items retained have been re-ordered so they are more aligned with the parallel oral hygiene items. See **S1: OHBSS-SF instrument** for the full set of instructions and complete measure and response options in both English and Spanish. The instructions, response options, optional items, and scoring instructions for the OHBSS-SF are the same as for the full-length OHBSS scales.

The nine full-length OHBSS scales assessed brushing social support from family, healthcare providers, and others/friends (abbreviated as BF, BP, BO); flossing social support from family, healthcare providers, and others/friends (FF, FP, FO); and dental care support from family, healthcare providers, and others/friends (DF, DP, DO, respectively). Respondents are asked to indicate the frequency of support received from family, healthcare providers, or others/friends. The 5-point response options ranged from never to always. Scores are averaged, and higher scores reflect more frequent receipt of social support. See **S2: full length OHBSS scales**.

Table 2 compares the contents of the full-length OHBSS and OHBSS-SF scales for each oral health behavior. The OHBSS-SF reduced the number of required items from 37 to 17. The set of seven optional items were unchanged, and could also be included with the OHBSS-SF.

Table 3 compares the types of social support in the full-length versus short-form versions of the scales. Appraisal social support is no longer captured in the OHBSS-SF. Both versions of the scales still predominantly reflect informational social support. The expert panel discussed the impact of these changes and the tradeoff in content coverage. The decisions for which items to retain were driven in part by the intended use in a future intervention, and which types of social support would be most critical to capture. Descriptive statistics for all the full-length items were examined and some different combinations of items were discussed, but there was consensus about the best set of items to retain if the scales were to be trimmed. The expert panel decided the reduction in length was beneficial, and to move forward with evaluating the psychometric properties of the OHBSS-SF.

### Measures

The same set of validated instruments used to demonstrate convergent and divergent validity with the full-length OHBSS scale were used again here to validate the OHBSS-SF (46). Table 4 summarizes these instruments, cites the source, and lists the Cronbach’s alpha observed in this study (in the full sample, and by language group).

### Self-reported Oral Health Behaviors

Three target behaviors were assessed via self-report. Oral hygiene behaviors were continuous counts of the frequency of brushing and flossing in the last week. Reported time since last dental care visit was dichotomized to reflect if the participant had a dental care visit within the last 12 months, for any reason.

### Self-reported Oral Health Status

Three oral health status indicators were assessed via self-report. Participants indicated if they thought they had gum disease. The number of missing teeth due to disease was originally captured as none, one to five, six or more but not all, or all, and were combined to reflect one or more but not all versus none. Tooth loss due to disease reflects worse oral health. Global oral health status and overall health of teeth and gums was originally asked to rate on a scale from poor, fair, good, very good, or excellent; responses were combined into fair/poor versus good/very good/excellent for this analysis.

### Subsample 1: Dental Exam data collection

Study dental exams were conducted by trained and calibrated dental staff, following a clinical protocol, detailed elsewhere (46). Briefly, this study employed a primarily visual exam and periodontal probing to yield tooth counts and clinically assessed oral health status indicators. Each present tooth in the mouth was recorded and evaluated. All clinical indices of interest were summarized based on 28 teeth (excluding third molars). Several clinical oral health indicators were assessed to examine the presence and severity of both dental caries (also called decay or cavities) and periodontal disease (also called gum disease).

A total of 41 Subsample 1 participants completed a dental exam. Given the small subsample size, only continuous clinical outcome measures were included in analyses. For dental caries, the number of teeth with any untreated decay was tabulated (the D component in the Decayed, Missing, and Filled Teeth (DMFT) index (68)). For periodontal disease, the proportion of tooth sites in the mouth that exhibited bleeding on probing was tabulated, with a higher proportion indicating worse periodontal health. Subsample 1 participants received a $20 gift card, dental kit, summary of exam findings, local dental resource list, and referral if needed.

### Subsample 2: Repeat Survey data collection

A total of 56 Subsample 2 participants completed the full-length OHBSS scales online a second time (producing repeat OHBSS-SF scores), two to six weeks after completing the survey the first time. Subsample 2 participants received a $20 gift card.

### Analysis

Spearman correlations between the full length OHBSS and OHBSS-SF were tabulated. Descriptive statistics (means, standard deviations) and psychometric properties were tabulated for the OHBSS-SF, in the full sample and both subsamples, and also by language preference (English or Spanish), sex (male or female), and marital status (partnered/married or single). Only the required set of OHBSS-SF items were included in all analyses. Two cases had a few missing OHBSS responses, and these values were imputed with regression imputation in the final analytic sample. All other variables had complete responses and no missing data.

Table 5 outlines all psychometric properties assessed for the nine OHBSS-SF scales. Data cleaning, descriptive statistics and all correlations were conducted in SAS (v9.4, SAS Institute, Cary, NC). Spearman correlations (r_s_) were calculated for all continuous scales. First, the study team expected convergent validity to be demonstrated as significant, high correlations (≥0.8) between the full length OHBSS and OHBSS-SF, given they are a subset of full length OHBSS items intended to measure the same construct.

Second, using MPLUS version 8, confirmatory factor analyses (CFAs) examined the structural validity of the OHBSS-SF scales. First, a set of CFAs were conducted in the full sample, then by study design subgroups (language preference, sex, and marital status). CFAs evaluated the brushing social support subscales’ three-factor structure of social support for brushing from family (BF-SF), healthcare providers (BP-SF), and others/friends (BO-SF). CFAs evaluated the fit of the flossing social support subscales’ three-factor structure of social support for flossing from family (FF-SF), healthcare providers (FP-SF), and others/friends (FO-SF). CFAs evaluated the fit of the dental care social support subscales’ three-factor structure of social support for dental care from family (DF-SF), healthcare providers (DP-SF), and others/friends (DO-SF).

All CFAs assessed the model fit for the OHBSS-SF factor structure following the same set of recommendations.(69) The Satorra-Bentler *χ*^2^ (S-B*χ*^2^) is robust in multivariate non-normal data and was used to evaluate statistical fit (70). S-B*χ*^2^ indicated adequate statistical fit if the estimate was non-significant at alpha 0.05. However, S-B*χ*^2^ can falsely reject an adequate model due to being sensitive to large sample sizes. To address this limitation, descriptive fit was also evaluated with the comparative fit index (CFI; ≥0.90 is adequate fit) (71), the root mean square error of approximation (RMSEA; ≤0.08 is adequate fit) (72), and the standardized root mean residual (SRMR; ≤0.08 is adequate fit) (73). The CFI, RMSEA, and SRMR together indicated overall adequate descriptive fit if two or three of these estimates showcased adequate fit. An exploratory phase was planned for CFAs that did not find statistical fit and descriptive fit. The modification fit indices were examined to find possible changes to the model that could help achieve adequate fit. The study team implemented changes that were theoretically reasonable and practical, including allowing items from a similar scale to correlate. Findings of acceptable statistical and/or descriptive fit were considered as evidence for structural validity in the corresponding set of OHBSS-SF scales, in the full sample, and for subgroups (by language, sex, and marital status).

Internal consistency statistics were examined for the OHBSS-SF scales in the full sample, and also by language, sex, and marital status subgroups. R studio was used to calculate (v4.0.3 (2010-10-10) Boston, MA) Cronbach’s alphas and McDonald’s omegas and bootstrapped 95% confidence intervals (CIs) (74, 75). Both alphas and omegas were tabulated to address potential biases in only reporting alphas (76). Higher alphas and omegas reflect greater internal consistency, with ≥0.7 as a minimally accepted threshold (74, 75, 77).

Spearman correlations (r_s_) were employed to assess convergent and divergent validity of the OHBSS-SF with other validated social support scales. Analyses explored the OHBSS-SF scales in the full sample, and also by language, sex, and marital status subgroups, using SPSS 22 and SAS v9. The study team expected significant, moderate correlations (0.3–0.5 range) between OHBSS-SF and the three other validated social support scales (ISEL-12, mMOS, MSPSS and associated subscales). Similar to the patterns observed with the full length OHBSS scales, patterns of higher correlations were expected with source of social support groups that aligned between the OHBSS-SF and other social support scales (for example, the others/friends source of support group in OHBSS-SF was expected to correlate more highly with the friends group in the MSPSS). No significant correlations or only weak (≤0.3) significant correlations with SASH and MDAS were expected.

Spearman correlations (r_s_) for continuous measures and ANOVAs for categorical variables were used to examine the associations between the OHBSS-SF and oral health behaviors and outcomes to explore predictive validity. Patterns of higher correlations and associations were expected in the weak to moderate range with oral health behaviors, as proximal behaviors of interest represented in each OHBSS-SF scale. Significant, positive associations were expected between the scales and corresponding behavior. Weak associations were expected between the OHBSS-SF scales and oral health indicators, given the hypothesized distal relationship between social support and indicators (both self-reported from the full sample and clinically assessed from Subsample 1). These analyses examined the OHBSS-SF scales in the full sample, and also by language preference, sex, and marital status.

In Subsample 2 with two time points (n=56), test-retest reliability was examined for the OHBSS-SF scales in the full sample, and also by language preference, sex, and marital status subgroups. R studio (v4.0.3 (2010-10-10) Boston, MA) was used to calculate intraclass correlation coefficients (ICC) estimates and their 95% confident intervals for a design of repeated surveys, based on a single measure, absolute agreement, and a two-way mixed-effects model (78). ICCs between 0.60 to 0.74 were considered “good,” and higher was “excellent” (79).

## RESULTS

Table 6 summarizes the characteristics of the full sample and subsamples. In the full sample, 60% of participants completed the survey in English, 21% were male, and 37% were married.

Table 7 summarizes the OHBSS-SF descriptives. For a more detailed breakdown of OHBSS-SF scale scores (mean, SD, median, mode, skewness and kurtosis, see **S3: OHBSS-SF detailed distributions**). Overall, there was a pattern of social support from healthcare providers had the highest mean score, indicative of more social support from that group. This pattern was consistent in the full sample, and by language preference, sex, and marital status. There were significant group differences by language for these social support scales: BF-SF, FF-SF, DF-SF, and DP-SF. There were significant group differences by sex for these social support scales: BP-SF, FP-SF, and DP-SF. There were significant group differences by marital status for the FF-SF scale.

Table 8 summarizes the correlations between the full length OHBSS and OHBSS-SF in the full sample and by subgroups. There were strong correlations between the corresponding full-length and OHBSS-SF versions of the subscales, ranging from r_s_=0.95 (DP-SF and DP) to r_s_=0.99 (BF-SF and BF) in the full sample. This pattern of correlations supports the use of the OHBSS-SF. All correlations were statistically significant.

### Confirmatory Factor Analysis Results

**Figure 1.**
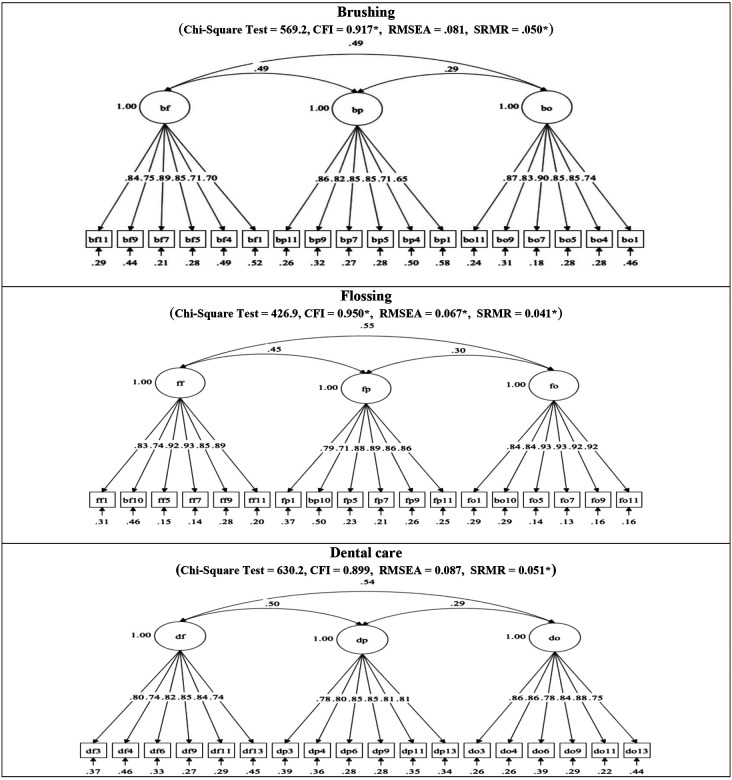
Factor structures for the OHBSS-SF brushing, flossing, and dental care social support scales in the full sample. Full-length OHBSS item numbers are shown for the OHBSS-SF items. ^1^Flossing social support scale short form has 6 items, including the one general hygiene item that is also in the Brushing social support scale. ^2^Sources of Support: F=Family, P=Providers, O=Others/Friends. CFI=Comparative Fit Index; RMSEA=Root Mean Square Error of Approximation; SRMR=Standardized Root Mean Square Residual.

Figure 1 displays the factor structures for the OHBSS-SF scales in the full sample for brushing social support, flossing social support, and dental care social support. The three factors represent the three different sources of support groups: family, healthcare providers, and others/friends.

#### Brushing social support

CFAs support the structural validity of the OHBSS-SF scales in the full sample and by language preference, sex, and marital status. The brushing social support subscales have adequate structural validity for a three-factor structure of social support for brushing from family (BF-SF), healthcare providers (BP-SF), and others/friends (BO-SF). The three-factor structure had adequate descriptive fit in the full sample (CFI=0.917, RMSEA=0.081, SRMR=0.050), as well as in the English-speaking subgroup (CFI=0.930, RMSEA=0.077, SRMR=0.053), female subgroup (CFI=0.915, RMSEA=0.082, SRMR=0.052), and married subgroup (CFI=0.915, RMSEA=0.082, SRMR=0.058).

The three-factor structure needed slight changes to reach adequate descriptive fit in the Spanish-speaking and male subgroups. In the Spanish-speaking subgroup, adequate fit was achieved only after the BF1 item and BF7 were respectively allowed to correlate with the BP1 item and BP7 item (CFI=0.902, RMSEA=0.086, SRMR=0.059). In the male subgroup, adequate fit was achieved only after the BF1 item was allowed to correlate with the BP1 item (CFI=0.919, RMSEA=0.083, SRMR=0.072). Allowing these items to correlate is appropriate. These items have overlapping variance, considering that they are from overlapping subscales or are from the same subscales. As expected, the three-factor structure had poor statistical fit in the full sample and in all subgroups. Despite the slight changes in certain subgroups and poor statistical fit, finding descriptive fit is evidence for the brushing social support subscales’ structural validity of their three-factor structure (i.e., BF-SF, BP-SF, BO-SF) in the full sample (Figure 1) and for subgroups, by language preference (Table 9; Figure 2a), sex (Table 10; Figure 3a), and marital status (Table 11; Figure 4a).

#### Flossing social support

The flossing social support subscales have adequate structural validity for a three-factor structure of social support for flossing from family (FF-SF), healthcare providers (FP-SF), and others/friends (FO-SF). The three-factor structure had adequate descriptive fit in the full sample (CFI=0.950, RMSEA=0.067, SRMR=0.041), Spanish-speaking subgroup (CFI=0.957, RMSEA=0.063, SRMR=0.042), English-speaking subgroup (CFI=0.936, RMSEA=0.078, SRMR=0.048), male subgroup (CFI=0.922, RMSEA=0.086, SRMR=0.047), female subgroup (CFI=0.945, RMSEA=0.071, SRMR=0.051), single subgroup (CFI=0.945, RMSEA=0.069, SRMR=0.046), and married subgroup (CFI =0.957, RMSEA=0.065, SRMR=0.051). No changes were needed to achieve adequate descriptive fit in any subgroup. As expected, this three-factor structure had poor statistical fit in the full sample and in all subgroups. Despite the poor statistical fit, finding descriptive fit is evidence for the flossing social support subscales’ structural validity of their three-factor structure (i.e., FF-SF, FP-SF, FO-SF) in the full sample (Figure 1) and by language preference (Table 9; Figure 2b), sex (Table 10; Figure 3b), and marital status (Table 11; Figure 4b).

#### Dental care social support

The dental care social support subscales had adequate structural validity for a three-factor structure of social support for dental care from family (DF-SF), health providers (DP-SF), and others/friends (DO-SF). This three-factor structure had adequate descriptive fit only in the female subgroup (CFI=0.904, RMSEA=0.084, SRMR=0.052) and married subgroup (CFI=0.903, RMSEA=0.089, SRMR=0.060).

The three-factor structure needed slight changes to reach adequate descriptive fit in the full sample and other subgroups. After the DF4 item and DO3 were respectively allowed to correlate with the DF11 item and DO4 item, adequate descriptive fit was achieved in the Spanish-speaking subgroup (CFI=0.906, RMSEA=0.093, SRMR=0.062), English-speaking subgroup (CFI=0.909, RMSEA=0.083, SRMR=0.052), single subgroup (CFI=0.908, RMSEA=0.085, SRMR=0.052), and full sample (CFI=0.916, RMSEA=0.080, SRMR=0.050). In the male subgroup (CFI=0.901, RMSEA=0.098, SRMR=0.066), adequate descriptive fit was achieved after the following items were allowed to correlate: DF6 with DF4; DF13 with DF4; DP6 with DP3; DP13 with DP4 and DP9; DO4 with DO3; and DO13 with DO9. Allowing these items to correlate is reasonable. These items have overlapping variance, considering that they come from overlapping subscales or are from the same subscales. As expected, the three-factor structure had poor statistical fit in the full sample and in all subgroups. Despite the slight changes and poor statistical fit, finding descriptive fit is evidence for the dental care social support subscales’ structural validity of their three-factor structure (i.e., DF, DP, DO) in the full sample (Figure 1) and by language preference (Table 9; Figure 2c), sex (Table 10; Figure 3c), and marital status (Table 11; Figure 4c).

### Internal Consistency Results

Table 12 summarizes the internal consistency for the OHBSS-SF scales in the full sample and by subgroup, and are evidence of the scales’ reliability. All subscales exhibited very high Cronbach’s alphas (α), with α≥0.85 in the full sample, α≥0.87 in the Spanish-speaking subgroup, α≥0.83 in the English-speaking subgroup, α≥0.87 in the male subgroup, α≥0.85 in the female subgroup, α≥0.86 in the single subgroup, and α≥0.84 in the married subgroup. Similarly, McDonald’s omegas (Ω) were also very high, with Ω≥0.86 in the full sample, Ω≥0.87 in the Spanish-speaking subgroup, Ω≥0.84 in the English-speaking subgroup, Ω≥0.89 in the male subgroup, Ω≥0.85 in the female subgroup, Ω≥0.86 in the single subgroup, and Ω≥0.85 in the married subgroup.

### Convergent Validity Results

The descriptive statistics for the three general social support scales for the full sample and by subgroup are summarized in Table 13. There were no significant differences by language subgroup. There were significant differences for ISEL and the ISEL-appraisal subscale, MSPSS and its subscales, and the mMOS by sex. There were significant differences for the ISEL and its subscales, MSPSS subscales and mMOS by marital status.

Table 14 presents the correlations between the OHBSS-SF scales and the general social support scales. In the full sample, the patterns of findings were generally consistent with expectations, with most pairings exhibiting weak significant correlations. Most of the significant correlations were between OHBSS-SF scales assessing social support from family and providers and most of the general social support scales and subscales; there were fewer correlations between OHBSS-SF social support from others/friends and general scales. Collectively, the patterns suggest social support assessed across all nine OHBSS-SF scales (BF-SF, BP-SF, FF-SF, FP-SF, DF-SF, DP-SF, and to a lesser degree BO-SF, FO-SF, DO-SF) correlate with existing validated general social support measures.

There were many significant correlations by language subgroup. Patterns of correlations differed by sex. Among men, there were fewer significant weak correlations, mostly observed with the OHBSS-SF social support from family subscales with general social support. Among women, most of the OHBSS-SF scales were weakly correlated with general social support scales. Patterns of correlations differed by marital status. Among single individuals, most of the OHBSS-SF scales were weakly correlated with general social support scales. However, among married individuals, there were few significant weak correlations, mostly with the OHBSS-SF dental care social support scales and general social support scales.

### Divergent Validity

Descriptives for the SASH and MDAS are summarized in Table 15. There were significant differences in scores across SASH and its subscales and the MDAS by language preference subgroups and marital status subgroups. SASH and MDAS scores were lower among the Spanish-speakers, and among those who were married.

There is evidence to support divergent validity of the final OHBSS-SF scales compared to the SASH and MDAS scales. A few OHBSS-SF scales were weakly correlated with SASH or MDAS. Generally, there were few significant correlations (see Table 16), which were very weak.

### Oral health behaviors and self-reported status

Table 17 summarizes the frequency of oral health behaviors in the past week, dental visit history in the prior year, and self-reported oral health status measures (fair/poor oral health, gum disease, missing any teeth due to disease). In the full sample, the mean brushing frequency was 12.2 times/week, flossing mean was 5.9 times/week, and 53% had visited the dentist within the last year. Slightly more than half rated their oral health as fair/poor, one-quarter reported having gum disease, and over one-third lost teeth because of disease. There were differences by sex in weekly reported flossing frequency and dental visits; women flossed more often than men, and were more likely to have had a recent dental visit than men. There were no significant differences by language preference or marital status.

Table 18 presents the correlations between the OHBSS-SF scales and brushing and flossing frequency. In the full sample, the patterns of findings were generally consistent with expectations, with some OHBSS-SF hygiene scales (BP-SF, FF-SF, FP-SF) weakly positively correlated with brushing (r_s_=0.09–0.14). All OHBSS-SF hygiene scales were weakly positively correlated with flossing (r_s_=0.09–0.23).

There were some different patterns of correlations between OHBSS-SF hygiene items and brushing and flossing across subgroups. Overall, there were fewer significant correlations with brushing frequency. There were two significant OHBSS-SF flossing item correlations with flossing among English-speakers (FF-SF and FO-SF; r_s_=0.14–0.16). Several OHBSS-SF hygiene items had significant correlations among Spanish-speakers with brushing (FF-SF, FP-SF; r_s_=0.15–0.22) and flossing (BP-SF, BO-SF, FF-SF, FP-SF, FO-SF; r_s_=0.14–0.32).

Among men, there were two significant OHBSS-SF item correlations with brushing (BF-SF and FF-SF; r_s_=0.31–0.33), and four significant correlations with flossing (BF-SF, BP-SF, FF-SF, FP-SF; r_s_=0.19–0.28). Among women, only FF-SF was significantly but weakly correlated with brushing (r_s_=0.10), and several OHBSS-SF hygiene items correlated with flossing (BP-SF, BO-SF, FF-SF, FP-SF, FO-SF; r_s_=0.10–0.22).

Among single individuals, there was one item significantly but weakly correlated with brushing (FP-SF; r_s_=0.12), and four significant but weakly correlations with flossing (B0-SF, FF-SF, FP-SF, FO-SF; r_s_=0.12–0.22). Among married individuals, only FF-SF was significantly but weakly correlated with brushing (r_s_=0.18), and several OHBSS-SF hygiene items correlated with flossing (BP-SF, FF-SF, FP-SF, FO-SF; r_s_=0.19–0.28).

Dental care social support scale scores (DF, DP, DO) by recent dental visit showed significant differences for the healthcare provider source of support group, in the full sample, among English-speakers, females, and single individuals (see Table 19). Those subgroups of participants with a dental visit in the last year had higher dental care social support scores from healthcare providers than those without a visit in the past year.

Dental care social support scale scores (DF-SF, DP-SF, DO-SF) by self-reported periodontal disease status were generally higher on average among those individuals without disease compared to those with disease (see Table 20), but there was only one significant difference for the healthcare provider source of support group in the full sample (DP-SF).

Dental care social support scale scores (DF-SF, DP-SF, DO-SF) were generally higher on average among individuals with better self-rated oral health status compared to those with fair/poor self-rated status (see Table 21). Most subgroups exhibited similar patterns of significant differences, with higher social support scores (reflecting more frequent social support) among those with better self-rated oral health compared to those with fair/poor self-rated oral health.

Dental care social support scale scores (DF-SF, DP-SF, DO-SF) were similar across all groups for those with and without any missing teeth due to disease (see Table 22). There were no significant differences across any groups.

### Subsample 1: Predictive Validity

Table 23 summarizes the descriptive characteristics for the two clinical oral health measures from the 41 participants with dental exams. The OHBSS-SF scales were not correlated with either of the two clinical oral health indicators.

### Subsample 2: Test-retest reliability

Test-retest reliability of the OHBSS-SF was assessed with 56 individuals, and ICCs are presented in Table 24. Similar to the full length OHBSS scales, test-retest reliability was good overall in the full sample and better among Spanish-speakers than English-speakers. In Subsample 2, seven of the nine ICCs were 0.60 or above, and the remaining two ICCs were very close to 0.60. In Spanish, all nine ICCs were above 0.75, and in the “excellent” range. However, the OHBSS-SF scales exhibited lower ICCs in English, with only four of the nine scale ICCs in the “good” range.

Most of the ICCs were in the good-excellent end of the range for the OHBSS-SF scales in the subgroups for men and women with three exceptions. Among men, BF-SF and FF-SF ICCs were below the “good” threshold, and among women, FO-SF was below the “good” threshold.

For both marital status subgroups, there was a clear pattern of less than good ICCs for nearly all the OHBSS-SF from the Others/Friends source of support group. BF-SF among married individuals also had a lower ICC. The OHBSS-SF test-retest reliability was otherwise in the good-excellent end of the range for all other behavior-source combinations by marital status.

## DISCUSSION

This paper described the item reduction process to create the nine OHBSS-SF scales, a set of measures to assess oral health behavior-specific social support from different sources. Required items went from 37 in the full length version down to 17 in the shorter version, a 46% decrease in length. The OHBSS-SF could still produce nine distinct individual OHBSS-SF scales for combinations of three different behaviors from three different sources of support. While items in the OHBSS-SF reflect fewer types of social support, this shorter version correlates highly with the full-length version. Overall, the pattern of results supports that the OHBSS-SF has acceptable psychometric properties.

The model fit indices and psychometric properties for each of the nine OHBSS-SF scales were acceptable in the full sample. Minimal adjustments were needed to model to ascertain reasonable fit. There were some notable different patterns that emerged in the study design subgroup comparisons by language preference, sex, and marital status. Collectively, these results suggest that the OHBSS-SF scales demonstrate structural validity. Additionally, OHBSS-SF demonstrated internal reliability. This is not surprising, given that the retained items reflect the informational and instrumental types of social support that dominated the full-length scales; these similar items showed strong internal consistency.

Overall, the patterns of correlational results were as expected, and exhibited positive correlations with general validated social support measures in support of convergent validity. Also as expected, there were no or few weaker significant correlations with the validated dental anxiety and acculturation scales, supportive of divergent validity. There were some significant but weak correlations between the OHBSS-SF and self-reported oral health behaviors and measures, but not with the clinically assessed measures in the small Subsample 1. This pattern of results for predictive validity also mirrored the full-length scale results in this area, supporting the future use of the OHBSS-SF.

The OHBSS-SF scales were stable overall in the full sample and among Spanish-speakers, with good to excellent intra-class correlations, supporting test-retest reliability after two to six weeks. However, there were less consistent answers to OHBSS-SF provided over this time period, for English-speakers and for the subset of scales assessing support from Others/Friends among both the married and single subgroups. Some subgroups in Subsample 2 were small (<30 Spanish-speakers, men, married individuals), and larger sample sizes would be better for assessing ICCs for the test-retest reliability (80). There were only seven men in Subsample 2, so results should be interpreted with caution given the very small sample size.

The OHBSS-SF performed well, overall and by study design subgroup, though some significant differences by subgroup characteristics were identified. All the patterns were in expected directions, and balance by language was prioritized over achieving balance by sex or marital status. It is a study strength that our team accounted for these three study design factors to enable examining all psychometric properties stratified by these characteristics. The team purposefully enrolled enough participants in each subgroup to support these stratified analyses; these critical factors could strongly influence social support, and were important to assess in a new social support scale. The goal during recruitment efforts was to enroll a balanced sample by language, sex, and marital status. While sample size was adequate to support stratifying analyses to assess psychometric properties for all these subgroups, the split was not even, particularly by sex. Men and married individuals had the lowest number of participants across the subgroups of interest. The study team leveraged strong clinic partnerships and a range of community-based efforts in both English and Spanish throughout outreach, recruitment, enrollment, and offered support to complete participation. In-person, face to face recruitment in the community is a successful strategy for recruiting traditionally harder to reach Hispanic/Latinx participants in research. (81–85) A range of outreach and recruitment strategies were employed in this study, though less was done in-person during this post-COVID-19 pandemic study timeframe, making it more challenging to reach men and married individuals.

Although the present study added significantly to the literature on social support among Hispanic/Latinx group of Mexican origin, there are some study and methodological caveats that must be considered to place the value of our findings in an appropriate perspective. Language, sex, and marital status were each explored as dichotomies, and observed differences across these subgroups should be interpreted with that in mind. These demographic groupings do not reflect the full complexity of these participants’ lived experience in the California-Mexico border region. Future analyses can employ different modeling and analytic techniques to better examine how these demographics and other factors may interrelate with each other, oral health behaviors, social support and outcomes. Further, additional studies should explore the generalizability of the OHBSS-SF to adults belonging to other Hispanic/Latinx heritage sub-groups.

The OHBSS-SF scales are a useful, brief set of tools to quickly ascertain levels of oral health-context specific social support from three different sources. An additional benefit is that the subscales can be pulled out as needed by providers or researchers who may only be interested in certain behavior-source of support combinations. There are parallel items across the OHBSS-SF subscales, which enable comparisons across oral health behaviors. While the full-length measures were reduced substantially, the OHBSS-SF tools retained robust psychometric properties.

### Conclusion

This study provides evidence for the structural validity, internal consistency, convergent and divergent validity, predictive validity and test-retest reliability for the nine OHBSS-SF scales. The set of OHBSS-SF scales exhibited acceptable psychometric properties overall. This shorter version of these scales appears to have utility.

## Supplementary Material

S1: OHBSS-SF scales

S2: Full length OHBSS scales

S3: OHBSS-SF detailed distributions

This is a list of supplementary files associated with this preprint. Click to download.

- S1.OHBSSSFscales.pdf
- S2.FinalfulllengthOHBSSscales.pdf
- S3Tables.OHBSSSFdetaileddescriptives.pdf

## Figures and Tables

**Figure 2a.**
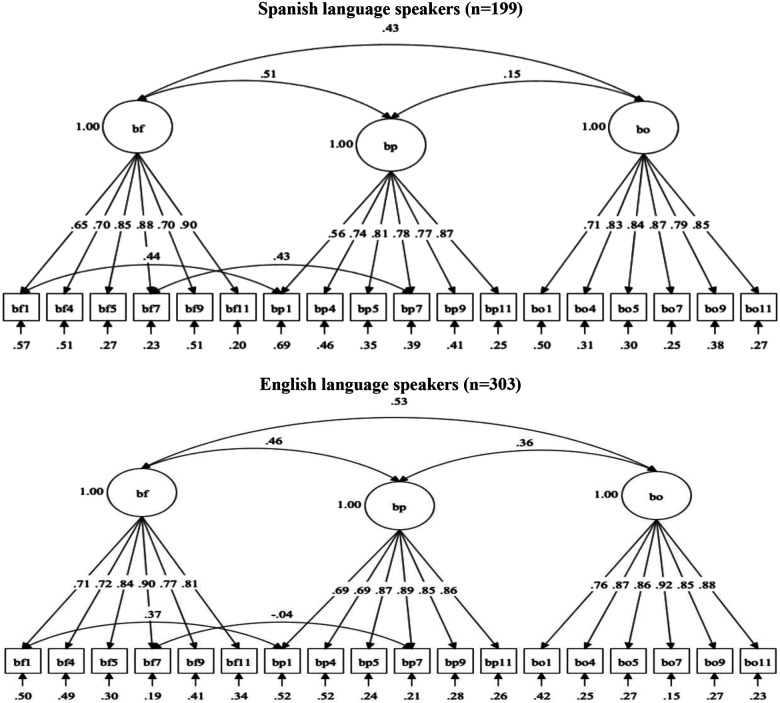
Three factor structure for the Brushing social support scales Short-Form (BF-SF, BP-SF, BO-SF), by language preference Full-length OHBSS item numbers are shown for the OHBSS-SF items. BF-SF=brushing social support from family short form; BP-SF=brushing social support from healthcare providers; BO-SF=brushing social support from others/friends.

**Figure 2b.**
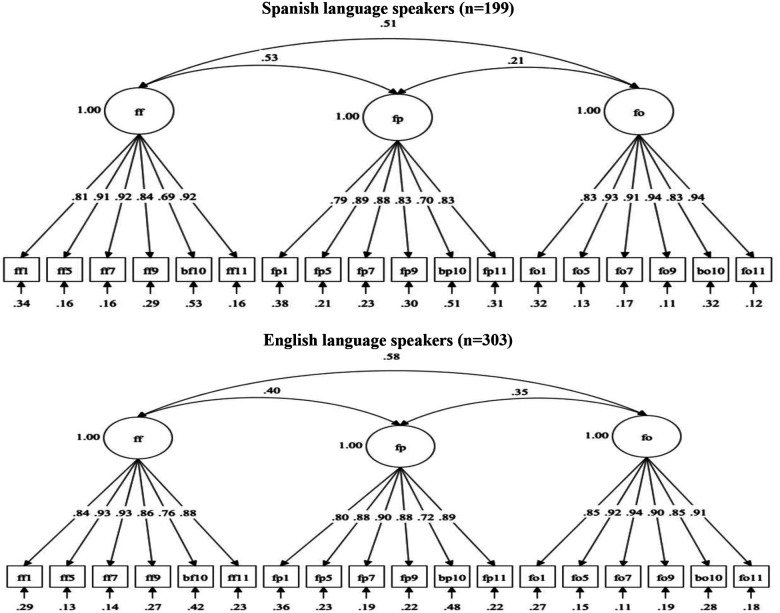
Three factor structure for Flossing social support scales, Short-Form (FF-SF, FP-SF, FO-SF), by language preference Full-length OHBSS item numbers are shown for the OHBSS-SF items. FF-SF=flossing social support from family (6-item short form); FP-SF=flossing social support from healthcare providers (6-item short form); FO-SF=flossing social support from others/friends (6-item short form). Flossing scale includes one item from the brushing social support scale.

**Figure 2c.**
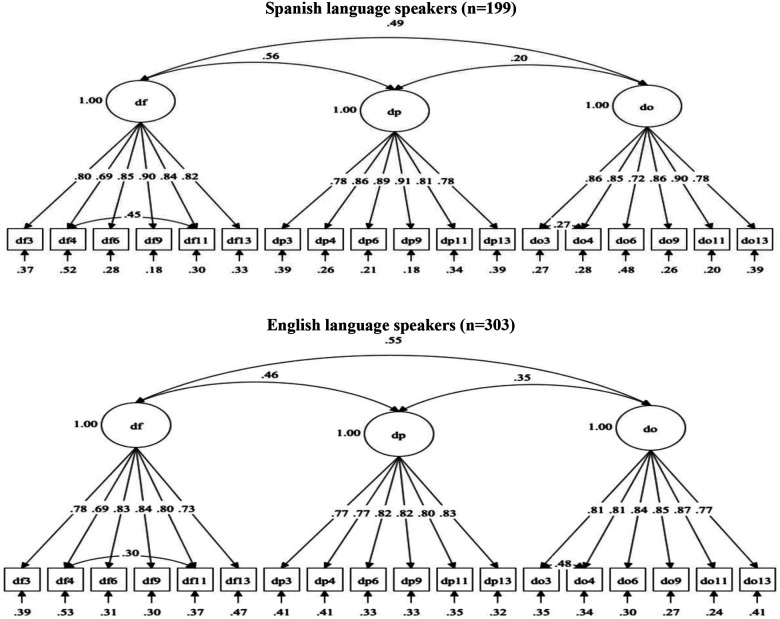
Three factor structure for Dental Care social support scales, Short-Form (DF-SF, DP-SF, DO-SF), by language preference Full-length OHBSS item numbers are shown for the OHBSS-SF items. DF-SF=dental care social support from family (6-item short form); DP-SF=dental care social support from healthcare providers (6-item short form); DO-SF=dental care social support from others/friends (6-item short form)

**Figure 3a.**
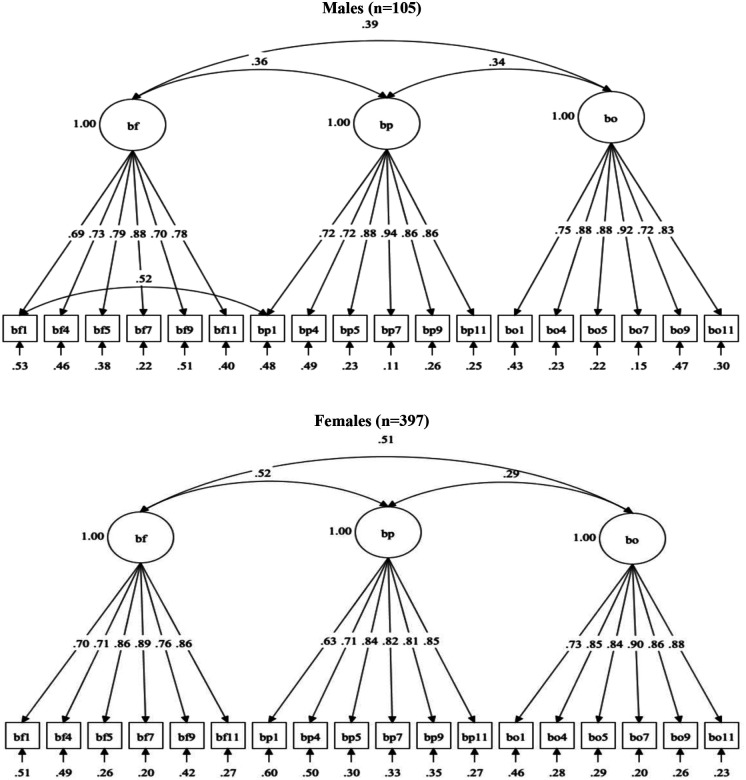
Three factor structure for final Brushing social support scales, Short-Form (BF-SF, BP-SF, BO-SF), by sex Full-length OHBSS item numbers are shown for the OHBSS-SF items. BF=brushing social support from family short form; BP=brushing social support from healthcare providers short form; BO=brushing social support from others/friends short form

**Figure 3b.**
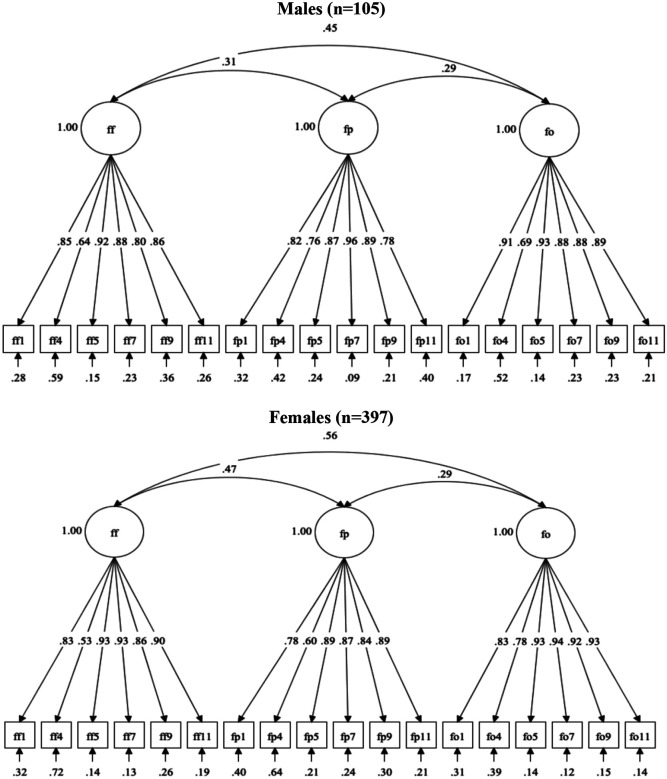
Factor structures for final Flossing social support scales Short Form (FF-SF, FP-SF, FO-SF), by sex Full-length OHBSS item numbers are shown for the OHBSS-SF items. FF-SF=flossing social support from family (6-item short form); FP-SF=flossing social support from healthcare providers (6-item short form); FO-SF=flossing social support from others/friends (6-item short form). One brushing item is scored with flossing subscale in the short form.

**Figure 3c.**
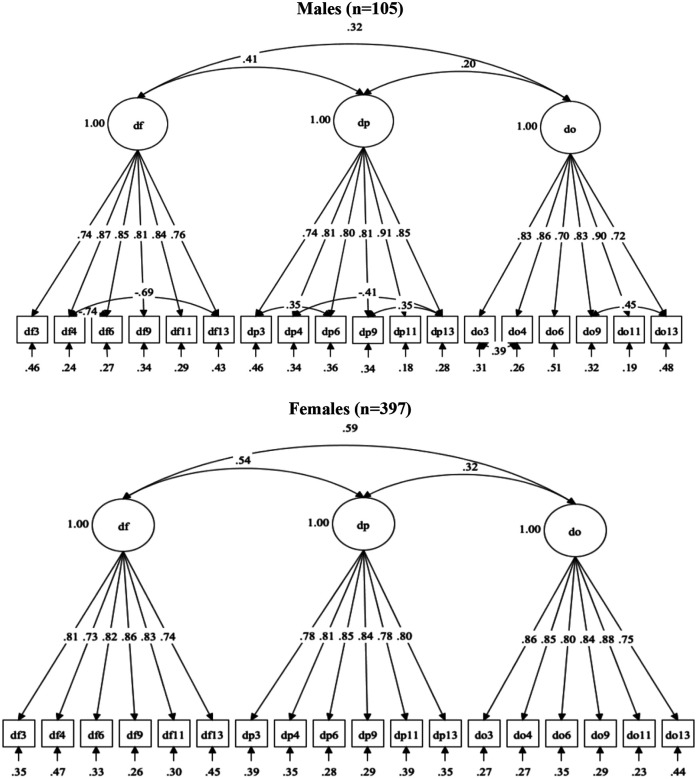
Factor structures for final Dental Care social support scales Short Form (DF-SF, DP-SF, DO-SF), by sex Full-length OHBSS item numbers are shown for the OHBSS-SF items. DF-SF=dental care social support from family (6-item short form); DP-SF=dental care social support from healthcare providers (6-item short form); DO-SF=dental care social support from others/friends (6-item short form)

**Figure 4a.**
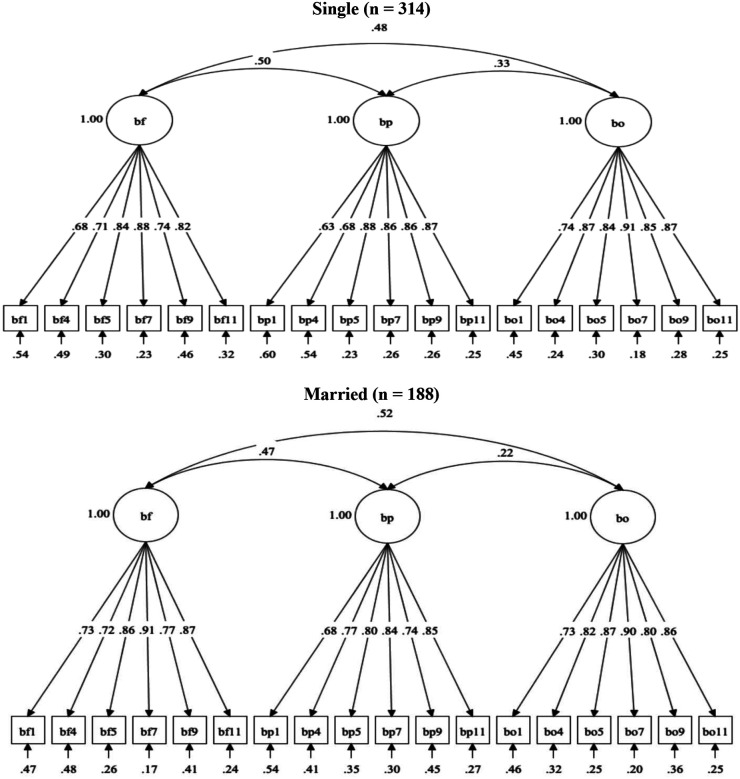
Three factor structure for final Brushing social support scales, Short-Form (BF-SF, BP-SF, BO-SF), by marital status Full-length OHBSS item numbers are shown for the OHBSS-SF items. BF-SF=brushing social support from family (6-item Short Form); BP-SF=brushing social support from healthcare providers, (6-item Short Form); BO=brushing social support from others/friends (6-item Short Form).

**Figure 4b.**
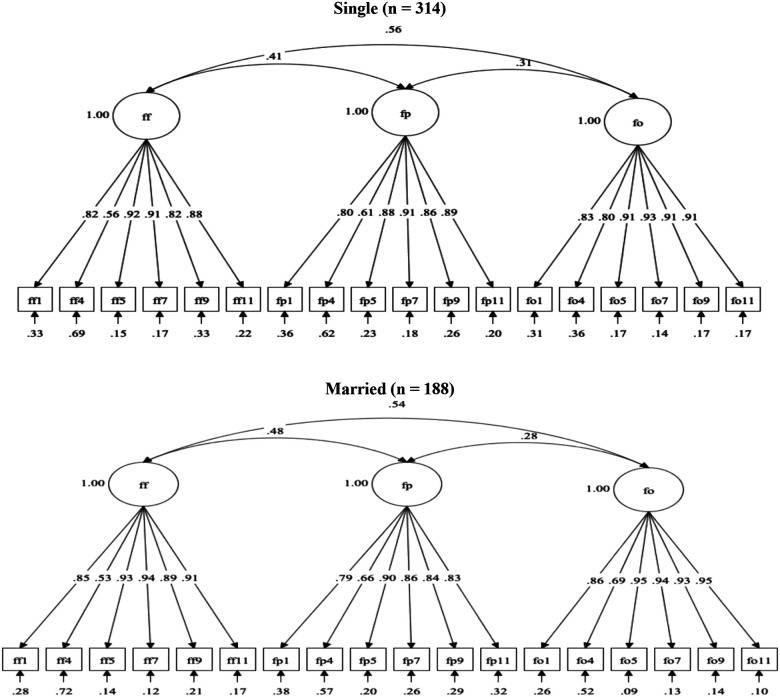
Factor structures for final Flossing social support scales Short Form (FF-SF, FP-SF, FO-SF), by marital status Full-length OHBSS item numbers are shown for the OHBSS-SF items. FF-SF=flossing social support from family (6-item SF); FP-SF=flossing social support from healthcare providers (6-item short SF); FO-SF=flossing social support from others/friends (6-item SF)

**Figure 4c.**
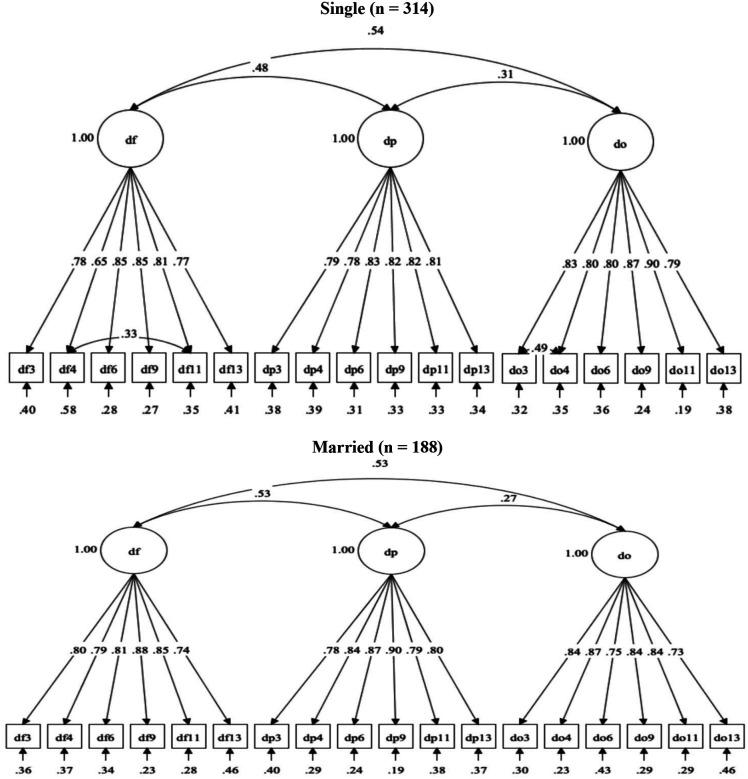
Factor structures for Dental Care social support scales Short Form (DF-SF, DP-SF, DO-SF), by marital status Full-length OHBSS item numbers are shown for the OHBSS-SF items. DF-SF=dental care social support from family (6-item SF); DP-SF=dental care social support from healthcare providers (6-item SF); DO-SF=dental care social support from others/friends (6-item SF)

**Table 1.** Oral Health Behavior Social Support Short-Form (OHBSS-SF) items in English and Spanish^1^

TYPE OF SOCIAL SUPPORT	BRUSHING (B) SOCIAL SUPPORT SCALE – SHORT FORM (SF)	Orig. item #	SF item
Instrumental	They show me how to brush my teeth. *Me enseñan a cepillarme los dientes*.^1^	B1	Ba
Informational^2^	They remind me to get more dental supplies (for example, toothbrush, toothpaste, floss, etc.). *Me recuerdan de conseguir más productos dentales (por ejemplo, cepillo, pasta, hilo dental, etc.)*	B4	Bb
Informational	They tell me that brushing my teeth is important to my health. *Me dicen que cepillarme los dientes es importante para mi salud*.	B5	Bc
Informational	They tell me to brush my teeth regularly (at least twice a day). *Me dicen que me cepille los dientes regularmente (al menos dos veces al día)*.	B7	Bd
Informational	They explain to me why my gums might bleed during or after brushing my teeth. *Me explican porque me pueden sangrar las encías durante o después de cepillarme los dientes*.	B9	Be
Emotional	They encourage me to brush my teeth. *Me motivan a cepillarme los dientes*.	B11	Bf
	FLOSSING (F) SOCIAL SUPPORT SCALE – SHORT FORM		
Instrumental	They show me how to floss my teeth. *Me enseñan a usar hilo dental*.	F1	Fa
Informational^2^	They remind me to get more dental supplies (for example, toothbrush, toothpaste, floss, etc.). *Me recuerdan de conseguir más productos dentales (por ejemplo, cepillo, pasta, hilo dental, etc.)*	F4	Fb
Informational	They tell me that flossing my teeth is important to my health. *Me dicen que usar hilo dental es importante para mi salud*.	F5	Fc
Informational	They tell me to floss my teeth regularly (at least once a day). *Me dicen que use hilo dental regularmente (al menos una vez al día)*.	F7	Fd
Informational	They explain to me why my gums might bleed during or after I use floss. *Me explican porque me pueden sangrar las encías durante o después de usar hilo dental*.	F9	Fe
Emotional	They encourage me to floss my teeth. *Me motivan a usar hilo dental*.	F11	Ff
	DENTAL CARE (D) SOCIAL SUPPORT SCALE – SHORT FORM		
Instrumental	They help me change how I take care of my teeth. *Me ayudan a cambiar como me cuido los dientes*.	D11	Da
Informational	They remind me about my dental appointment. *Me recuerdan de mi cita dental*.	D3	Db
Informational	They tell me that going to the dentist is important to my health. *Me dicen que ir al dentista es importante para mi salud*.	D6	Dc
Informational	They tell me to go to the dentist regularly. *Me dicen que vaya al dentista de manera regular*.	D9	Dd
Informational	They tell me what will happen during my dental treatment. *Me dicen lo que pasará durante mi tratamiento dental*.	D4	De
Informational	They tell me to go to the dentist for my dental problems or discomfort. *Me dicen que vaya al dentista con respecto a mis problemas o molestias dentales*.	D13	Df

1 Spanish item italicized

2 This item is only asked once, but scored with both the brushing and flossing social support scales.

**Table 2.** Number of items in the full-length OHBSS scales and OHBSS-SF scales

Behavior	# items in required full-length OHBSS scales	# items in OHBSS-SF scales
REQUIRED ITEMS		
Brushing	12*	6*
Flossing	11*	5*
Dental Care	14	6
TOTAL REQUIRED	37**	17**
OPTIONAL ITEMS^1^		
Translation	1	1
Transportation	1	1
Payment	3	3
Worries	2	2
MAXIMUM ADDITIONAL OPTIONAL ITEMS^1^	7	7
TOTAL MAXIMUM REQUIRED AND OPTIONAL^1^ ITEMS	44**	24**

* 1 item that asked about social support for dental hygiene supplies is asked once, but is scored with both the Brushing and Flossing social support scales.

** Participants rate each item three times, once for each source of support group: 1) Family, 2) Healthcare Providers, 3) Others/Friends

1 Optional items are only asked if applicable. All items in the optional set are related to dental care access barriers. Optional items can be included in the full-length OHBSS scales and OHBSS-SF scales. Optional items are shown in supplemental materials (see **S1** and **S2**).

**Table 3:** Number of required items in the full-length OHBSS scales and OHBSS SF scales, by type of social support

Full-length OHBSS**					OHBSS-SF**				
Type of Social Support	Brush	Floss	Dental Care	TOTAL	Type of Social Support	Brush	Floss	Dental Care	TOTAL
Instrumental	3	3	5	11	Instrumental	1	1	1	3
Informational	7	6*	8	21	Informational	4	3*	5	12
Emotional	1	1	1	3	Emotional	1	1	0	2
Appraisal	1	1	0	2	Appraisal	0	0	0	0
TOTAL	12	11	14	37	TOTAL	6	5	6	17**

* 1 item that asked about social support for dental supplies is asked once, but is scored with both the Brushing and Flossing social support scales.

** Participants rate each item three times, once for each source of support:

1) Family, 2) Health Providers, 3) Others/Friends

**Table 4.** Validated instruments

Instrument Name	# items and alphas^1^	Description and response options	Sources
**NEW OHBSS-SF scales:** Brushing-Family (BF-SF) Brushing-Providers (BP-SF) Brushing-Others (BO-SF) Flossing-Family (FF-SF) Flossing-Providers (FP-SF) Flossing-Others (FO-SF) Dental Care-Family (DF-SF) Dental Care-Providers (DP-SF) Dental Care-Others (DO-SF) **Full length OHBSS scales:** BF BP BO FF FP FO DF DP DO	17 items^2^ (6 items Brushing; 5 items Flossing^2^; 6 items Dental Care); See Table 12 for Cronbach’s alphas and McDonald’s omegas 37 required items^2^ (12 items Brushing; 11 items Flossing; 14 items Dental Care); All scales Cronbach’s alpha>0.95, English>0.95, Spanish>0.95	OHBSS-SF scales include nine scales to assess social support from behavior-source combinations based on three behaviors (brushing, flossing, dental care: B, F, D) from three sources of support (family, providers, others: F, P, O) **Response options:** 0 (never) to 4 (always). Full length OHBSS scales include nine scales assessing social support from behavior-source combinations based on three behaviors (brushing, flossing, dental care: B, F, D) from three sources of support (family, providers, others: F, P, O) **Response options:** 0 (never) to 4 (always)	See OHBSS-SF scales in **S1**. ENGLISH & SPANISH: Finlayson, Garcia-Alcaraz et al, 2025; Finlayson, Malcarne et al, 2025 (45, 46). See full length OHBSS scales in **S2**.
**Validated general social support scales**			
Interpersonal Support Evaluation List (ISEL-12)	12 items; Cronbach’s alpha=0.86, English=0.87, Spanish=0.84	ISEL-12 assesses the availability of functional social support and includes subscales for appraisal, belonging, and tangible support **Response options:** 0 (definitely false) to 3 (definitely true)	ENGLISH: Cohen, Mermelstein et al, 1985 (51); SPANISH: Seeman 1996; Sacco, Casado et al, 2011; Kumar, Calvo et al, 2012, Merz, Roesch et al, 2014 (52–55)
Modified Medical Outcomes Study-Social Support Survey (mMOS)	8 items; Cronbach’s alpha=0.96, English=0.96, Spanish=0.96	mMOS assesses companionship types of support. **Response options:** 1 (none of the time) to 5 (all of the time)	ENGLISH: MOS: Sherborne and Stewart,1991 (56) mMOS: Moser, Stuck et al, 2012 (57) SPANISH: Gómez-Campelo, Pérez-Moreno et al. 2014 (58)
Multidimensional Scale of Perceived Social Support (MSPSS)	12 items; Cronbach’s alpha=0.96, English=0.95, Spanish=0.96	MSPSS assesses perceived available emotional social support from three sources (family, friend, and significant other) **Response options:** 1 (very strongly disagree) to 7 (very strongly agree)	ENGLISH: Zimet, Dahlem et al, 1988; Zimet, Powell et al, 1990; Dahlem 1991 (59–61); SPANISH: Edwards 2002 (62)
**Other validated scales**			
Modified Dental Anxiety Scale (MDAS)	5 items; Cronbach’s alpha=0.78; English=0.85; Spanish=0.74	MDAS assesses dental anxiety under different dental appointment scenarios. **Response options:** 1 (not anxious) to 5 (extremely anxious)	ENGLISH: Humphris, Freeman et al, 2000 (63); SPANISH: Coolidge, Hillstead et al 2010 (64)
Short Acculturation Scale for Hispanics (SASH)	10 items; Cronbach’s alpha=0.90; English=0.83, Spanish=0.83	SASH assesses preferences for language use and social interactions. **Response options:** 1 (Only Spanish) to 5 (Only English), or 1 (All Hispanic/Latinx) to 5 (All non-Hispanic/Latinx)	ENGLISH and SPANISH: Marin, Sabogal et al 1987; Marin and Gamba 1996; Ellison, Jandorf et al 2011 (65–67)

1 Cronbach’s alpha in this sample, overall, and by language

2 OHBSS-SF and OHBSS count only include required items, and do not include the seven optional Dental Care items.

One brushing item is also scored with the flossing scales.

* self-rated oral health status and number of missing teeth due to disease are part of the set of eight self-reported periodontal disease survey questions

**TABLE 5.** OHBSS-SF psychometric properties

Analyses	Sample^1^	Psychometric Scale Development Property *Key Measures involved in analysis with the OHBSS-SF*^2^ *scales italicized*
Correlations	Full Sample (N=502)	**Convergent validity**- Assess association of the *OHBSS-SF scales* with the full-length *OHBSS scales*
Confirmatory Factor Analysis	Full Sample (N=502)	**Structural validity** – Confirm model fit of identified domains and identify *OHBSS-SF scales* factor structure
Cronbach’s alpha and McDonald’s omega	Full Sample (N=502)	**Internal reliability**- Assess internal consistency and extent of inter-relatedness of items in the *OHBSS-SF scales*
Correlations	Full Sample (N=502)	**Convergent validity**- Assess association of *OHBSS-SF scales* with three existing, accepted validated social support scales (*ISEL-12, mMOS, MSPSS*)^3^
Correlations	Full Sample (N=502)	**Divergent validity**- *OHBSS-SF scales* should not correlate too highly with scales for different external constructs measuring acculturation and dental anxiety (*SASH, MDAS*)^4^
Correlations, ANOVAs	Full Sample (N=502)	**Predictive validity**- Assess ability of the *OHBSS-SF scales* to predict target health behaviors (*brushing, flossing, dental visit*) and self-reported oral health status (*self-rated oral health, gum disease*)
ANOVAs	Subsample 1 (n=41)	**Predictive validity**- Assess ability of the *OHBSS-SF scales* to predict clinically-assessed oral health status (*untreated decay, periodontal disease*)
Intraclass correlations (ICC)	Subsample 2 (n=56)	**Test-retest reliability**- Assess ability of *OHBSS-SF scales* to reproduce scores with the same participant on a repeat survey after a short time period (2–6 weeks), assuming conditions have not changed. Scores compared to *repeat OHBSS-SF* responses.

1 All analyses were also stratified by language preference, sex, and marital status

2 OHBSS-SF: Oral Health Behavior and Social Support-Short Form

3 ISEL-12: Interpersonal Support Evaluation List, and associated subscales mMOS: Modified Medical Outcomes Study-Social Support Survey, and associated subscales MSPSS: Multidimensional Scale of Perceived Social Support, and associated subscales

4 SASH: Short Acculturation Scale for Hispanics; MDAS: Modified Dental Anxiety Scale

**Table 6.** Full Sample and Subsample Characteristics by Language, Sex, and Marital Status, Count (Percent)

	Full Sample^a^ N=502	Subsample 1 Dental Exams^b^ n=41	Subsample 2 Repeat Surveys^c^ n=56
Language Preference			
Spanish	199 (40)	20 (49)	21 (37)
English	303 (60)	21 (51)	35 (63)
Sex			
Female	397 (79)	34 (83)	49 (87)
Male	105 (21)	7 (17)	7 (13)
Marital Status			
Married	188 (37)	19 (46)	23 (41)
Single	314 (63)	22 (54)	33 (59)

a Significant differences by language preference, sex, marital status (p<0.05)

b Significant differences by sex (p<0.05)

c Significant differences by language, sex (p<0.05)

**Table 7.** OHBSS-SF descriptives in the full sample and by language preference, sex, and marital status

OHBSS-SF scales	Full Sample N=502 mean±SD	English n=303 mean±SD	Spanish n=199 mean±SD	Females n=397 mean±SD	Males n=105 mean±SD	Married n=188 mean±SD	Single n=314 mean±SD
Brushing-Family (BF-SF)^1^	2.30±1.26	2.16±1.27	2.52±1.21	2.33±1.28	2.20±1.16	2.40±1.30	2.24±1.23
Brushing-Providers (BP-SF)^2^	2.89±1.11	2.84±1.17	2.97±1.03	2.94±1.08	2.70±1.21	2.94±1.07	2.86±1.14
Brushing-Others (BO-SF)	0.95±1.15	0.93±1.19	0.97±1.10	0.94±1.16	0.97±1.16	0.87±1.09	1.00±1.19
Flossing-Family (FF-SF)^1, 3^	1.93±1.30	1.82±1.31	2.09±1.26	1.97±1.32	1.75±1.19	2.09±1.36	1.83±1.26
Flossing-Providers (FP-SF)^2^	2.84±1.19	2.81±1.21	2.89±1.17	2.92±1.16	2.54±1.28	2.92±1.17	2.80±1.21
Flossing-Others (FO-SF)	0.77±1.11	0.78±1.15	0.75±1.05	0.79±1.14	0.68±1.00	0.68±1.05	0.82±1.14
Dental Care-Family (DF-SF)^1^	2.19±1.23	2.11±1.23	2.32±1.23	2.20±1.25	2.19±1.17	2.29±1.25	2.14±1.22
Dental Care-Providers (DP-SF)^1, 2^	3.00±1.12	2.91±1.14	3.12±1.08	3.06±1.09	2.77±1.19	3.05±1.10	2.96±1.13
Dental Care-Others (DO-SF)^2^	1.00±1.15	0.98±1.18	1.03±1.10	1.04±1.18	0.85±1.00	0.93±1.08	1.04±1.19

OHBSS-SF = Oral Health Behavior Social Support Short-Form scales (17 required items). SD = standard deviation.

Response Options: 0=never, 1=rarely, 2=sometimes, 3=often, 4=always; higher mean subscale scores correspond to more frequent receipt of social support for that behavior (B=brushing, F=flossing, D=dental care) from that source (F=family, P=health providers, O=others/friends)

1 = significant group differences by language;

2= significant group differences by sex;

3= significant group differences by marital status

**Table 8.** Spearman Correlations between the OHBSS-SF and full length OHBSS

	Full length OHBSS, full sample and by language preference											
OHBSS-SF	FULL SAMPLE (N=502)			ENGLISH (n=303)			SPANISH (n=199)					
**BRUSHING**	**BF**	**BP**	**BO**	**BF**	**BP**	**BO**	**BF**	**BP**	**BO**			
BF-SF	0.98	0.51	0.47	0.98	0.48	0.50	0.98	0.49	0.41			
BP-SF	0.48	0.96	0.30	0.45	0.98	0.38	0.48	0.98	0.16			
BO-SF	0.48	0.32	0.98	0.52	0.38	0.99	0.43	0.20	0.99			
**FLOSSING**	**FF**	**FP**	**FO**	**FF**	**FP**	**FO**	**FF**	**FP**	**FO**			
FF-SF	0.99	0.49	0.50	0.98	0.43	0.56	0.99	0.56	0.49			
FP-SF	0.46	0.97	0.28	0.41	0.98	0.36	0.51	0.98	0.21			
FO-SF	0.51	0.32	0.98	0.57	0.38	0.99	0.51	0.25	0.99			
**DENT CARE**	**DF**	**DP**	**DO**	**DF**	**DP**	**DO**	**DF**	**DP**	**DO**			
DF-SF	0.98	0.54	0.50	0.98	0.46	0.53	0.98	0.57	0.51			
DP-SF	0.48	0.95	0.28	0.41	0.98	0.32	0.52	0.98	0.23			
DO-SF	0.50	0.30	0.98	0.53	0.36	0.99	0.51	0.26	0.98			
	Full length OHBSS, by sex						Full length OHBSS, by marital status					
OHBSS-SF	FEMALES (n=397)			MALES (n=105)			MARRIED (n=188)			SINGLE (n=314)		
**BRUSHING**	**BF**	**BP**	**BO**	**BF**	**BP**	**BO**	**BF**	**BP**	**BO**	**BF**	**BP**	**BO**
BF-SF	0.98	0.52	0.48	0.98	0.39	0.41	0.99	0.51	0.48	0.98	0.48	0.47
BP-SF	0.50	0.98	0.30	0.34	0.98	0.33	0.48	0.98	0.23	0.45	0.98	0.35
BO-SF	0.50	0.32	0.99	0.42	0.33	0.99	0.50	0.25	0.99	0.48	0.35	0.99
**FLOSSING**	**FF**	**FP**	**FO**	**FF**	**FP**	**FO**	**FF**	**FP**	**FO**	**FF**	**FP**	**FO**
FF-SF	0.99	0.52	0.55	0.98	0.30	0.44	0.99	0.54	0.53	0.98	0.44	0.55
FP-SF	0.49	0.98	0.31	0.27	0.98	0.28	0.490	0.98	0.28	0.42	0.98	0.32
FO-SF	0.56	0.34	0.99	0.42	0.30	0.98	0.53	0.31	0.99	0.56	0.35	0.99
**DENT CARE**	**DF**	**DP**	**DO**	**DF**	**DP**	**DO**	**DF**	**DP**	**DO**	**DF**	**DP**	**DO**
DF-SF	0.98	0.54	0.57	0.98	0.42	0.30	0.98	0.55	0.51	0.98	0.48	0.53
DP-SF	0.49	0.97	0.32	0.35	0.98	0.18	0.51	0.98	0.29	0.43	0.97	0.30
DO-SF	0.57	0.35	0.99	0.29	0.19	0.99	0.52	0.33	0.99	0.53	0.33	0.99

All correlations <0.0001;

Shaded cells=correlations between corresponding OHBSS-SF and full length OHBSS subscales

**TABLE 9.** Confirmatory Factor Analysis Model Fit Indices for OHBSS-SF scales, full sample and by language preference

	Chi-Square Test	CFI	RMSEA	SRMR	Minimal revisions to achieve acceptable model fit.^2^
Full sample (N = 502)					
**1. Three-factor Brush**	**569.2**	**0.917** *	**0.081**	**0.050** *	
**2. Three-factor Brush**	**497.1**	**0.930** *	**0.075** *	**0.048** *	**See changes below**
**1. Three-factor Floss** ^1^	**426.8**	**0.950** *	**0.067**	**0.041** *	
1. Three-factor Dental Care	630.2	0.899	0.087	0.051*	
**2. Three-factor Dental Care update**^1^ **(minor revisions)**	**546.0**	**0.916** *	**0.080**	**0.050** *	**See changes below**
Spanish (n = 199)					
1. Three-factor Brush	373.1	0.877	0.096	0.062*	
**2. Three-factor Brush**	**322.1**	**0.902** *	**0.086**	**0.059** *	**BF1 WITH BP1;** **BF7 WITH BP7;**
**1. Three-factor Floss** ^1^	**234.9**	**0.957** *	**.063** *	**0.042** *	
1. Three-factor Dental Care^2^	380.5	0.896	0.097	0.064*	
**2. Three-factor Dental Care**	**352.6**	**0.906** *	**0.093**	**0.062** *	**DF4 WITH DF11;** **DO3 WITH DO4;**
English (n = 303)					
**1. Three-factor Brush**	**369.5**	**0.930** *	**0.077** *	**0.053** *	
**2. Three-factor Brush**	**334.4**	**0.940** *	**0.072** *	**0.051** *	**BF1 WITH BP1;** **BF7 WITH BP7;**
**1. Three-factor Floss** ^1^	**376.7**	**0.936** *	**0.078** *	**0.048** *	
1. Three-factor Dental Care^2^	455.0	0.890	0.090	0.053*	
**2. Three-factor Dental Care update**	**399.3**	**0.909** *	**0.083**	**0.052** *	**DF4 WITH DF11;** **DO3 WITH DO4;**

Notes

1 Flossing social support scale short form has 6 items, including the one general hygiene item that is also in the Brushing social support scale.

2 Item abbreviations: B=Brushing, D=Dental Care; F=Family, P=Providers, O=Others/Friends.

* indicates fit indices that show at least acceptable model fit.

CFI = Comparative fit index;

RMSEA = Root Mean Square Error of Approximation;

SRMR = Standardized Root Mean Square Residual.

CFI values of at least 0.90 indicate acceptable model fit and values > 0.95 indicate good model fit.

SRMR and RMSEA values less or equal to 0.08 indicate acceptable model fit and values lower or equal to 0.05 indicate good model fit. Two out of the three descriptive fit indices need to show at least acceptable model fit to declare that a model has adequate descriptive model fit.

**Bolded** indicates acceptable model fit.

**TABLE 10.** Confirmatory Factor Analysis Model Fit Indices for OHBSS-SF scales, full sample and by sex

	Chi-Square Test	CFI	RMSEA	SRMR	Minimal revisions to achieve acceptable model fit.^2^
Full sample (N = 502)					
**1. Three-factor Brush**	**569.2**	**0.917** *	**0.081**	**0.050** *	
**2. Three-factor Brush**	**503.5**	**0.929** *	**0.075** *	**0.048** *	**See changes below**
**1. Three-factor Floss** ^1^	**426.9**	**0.950** *	**0.067** *	**0.041** *	
1. Three-factor Dental Care	630.2	0.899	0.087	0.051*	
**2. Three-factor Dental Care update**^1^ **(minor revisions)**	**511.2**	**0.922** *	**0.078** *	**0.050** *	**See changes below**
Males (n = 105)					
1. Three-factor Brush	251.1	0.898	0.093	0.074*	
**2. Three-factor Brush**	**225.4**	**0.919** *	**0.083**	**0.072** *	**BF1 WITH BP1;**
**1. Three-factor Floss** ^1^	**235.6**	**0.922** *	**0.086**	**0.047** *	
1. Three-factor Dental Care	341.5	0.835	0.123	0.071*	
**2. Three-factor Dental Care**	**250.4**	**0.901** *	**0.098**	**0.066** *	**DF6 WITH DF4;** **DF13 WITH DF4;** **DP6 WITH DP3;** **DP13 WITH DP4;** **DP13 WITH DP9;** **DO4 WITH DO3;** **DO13 WITH DO9;**
Females (n = 397)					
**1. Three-factor Brush**	**488.6**	**0.915** *	**0.082**	**0.052** *	
**1. Three-factor Floss** ^1^	**394.7**	**0.945** *	**0.071** *	**0.051** *	
1. Three-factor Dental Care	**506.1**	**0.904** *	**0.084**	**0.052** *	

Notes

1 Flossing social support scale short form has 6 items, includes the one general hygiene item that is also in the Brushing social support scale.

2 Item abbreviations: B=Brushing, D=Dental Care; F=Family, P=Providers; O=Others/Friends.

* indicates fit indices that show at least acceptable model fit.

CFI = Comparative fit index;

RMSEA = Root Mean Square Error of Approximation;

SRMR = Standardized Root Mean Square Residual.

CFI values of at least 0.90 indicate acceptable model fit and values > 0.95 indicate good model fit.

SRMR and RMSEA values less or equal to 0.08 indicate acceptable model fit and values lower or equal to 0.05 indicate good model fit. Two out of the three descriptive fit indices need to show at least acceptable model fit to declare that a model has adequate descriptive model fit.

**Bolded** indicates acceptable model fit.

**TABLE 11.** Confirmatory Factor Analysis Model Fit Indices for OHBSS-SF scales, full sample and by marital status

	Chi-Square Test	CFI	RMSEA	SRMR	Minimal revisions to achieve acceptable model fit.^2^
Full sample (N = 502)					
**1. Three-factor Brush**	**569.2**	**0.917** *	**0.081**	**0.050** *	
**1. Three-factor Floss** ^1^	**426.9**	**0.950** *	**0.067** *	**0.041** *	
1. Three-factor Dental Care	630.2	0.899	0.087	0.051*	
**2. Three-factor Dental Care update (minor revisions)**	**546.0**	**0.916** *	**0.080** *	**0.050** *	**See changes below**
Single (n = 314)					
**1. Three-factor Brush**	**455.1**	**0.907** *	**0.088**	**0.054** *	
**1. Three-factor Floss** ^1^	**330.1**	**0.945** *	**0.069** *	**0.046** *	
1. Three-factor Dental Care^2^	486.8	0.888	0.093	0.053*	
**2. Three-factor Dental Care**	**422.1**	**0.908** *	**0.085**	**0.052** *	**DF4 WITH DF11;** **DO3 WITH DO4;**
Married (n = 188)					
**1. Three-factor Brush**	**298.8**	**0.915** *	**0.082**	**0.058** *	
**1. Three-factor Floss** ^1^	**238.1**	**0.957** *	**0.065** *	**0.051** *	
**1. Dental Care** ^2^	**326.6**	**0.903** *	**0.089**	**0.060** *	

Notes

1 Flossing social support short form scale has 6 items, includes the one general hygiene item that is also in the Brushing social support scale short form.

2 Item abbreviations: D=Dental Care; F=Family, P=Providers, O=Others/Friends.

* indicates fit indices that show at least acceptable model fit.

CFI = Comparative fit index;

RMSEA = Root Mean Square Error of Approximation;

SRMR = Standardized Root Mean Square Residual.

CFI values of at least 0.90 indicate acceptable model fit and values > 0.95 indicate good model fit.

SRMR and RMSEA values less or equal to 0.08 indicate acceptable model fit and values lower or equal to 0.05 indicate good model fit. Two out of the three descriptive fit indices need to show at least acceptable model fit to declare that a model has adequate descriptive model fit.

**Bolded** indicates acceptable model fit.

**Table 12.** OHBSS-SF internal consistency for full sample and by language preference, sex, and marital status

	Cronbach’s Alpha (95% Confidence Interval)			McDonald’s Omega (95% Confidence Interval)		
	Family (F)	Provider (P)	Other (O)	Family (F)	Provider (P)	Other (O)
**Full Sample (N=502)**						
Brushing-SF	0.91 (0.89, 0.92)	0.86 (0.83, 0.88)	0.93 (0.92, 0.95)	0.91 (0.89, 0.92)	0.86 (0.84, 0.88)	0.94 (0.93, 0.95)
Flossing-SF	0.93 (0.92, 0.94)	0.87 (0.85, 0.89)	0.96 (0.94, 0.96)	0.93 (0.93, 0.94)	0.88 (0.86, 0.90)	0.96 (0.94, 0.96)
Dental Care-SF	0.91 (0.90, 0.92)	0.92 (0.90, 0.94)	0.93 (0.91, 0.94)	0.91 (0.90, 0.92)	0.92 (0.90, 0.94)	0.93 (0.91, 0.94)
**Language Preference**						
**Spanish language speakers (n = 199)**						
Brushing-SF	0.91 (0.89, 0.93)	0.87 (0.84, 0.89)	0.94 (0.92, 0.95)	0.91 (0.89, 0.93)	0.87 (0.85, 0.89)	0.94 (0.93, 0.96)
Flossing-SF	0.93 (0.92, 0.95)	0.87 (0.85, 0.90)	0.96 (0.94, 0.97)	0.94 (0.92, 0.95)	0.88 (0.86, 0.90)	0.96 (0.94, 0.97)
Dental Care-SF	0.90 (0.88, 0.92)	0.91 (0.89, 0.93)	0.93 (0.91, 0.94)	0.91 (0.88, 0.92)	0.91 (0.89, 0.93)	0.93 (0.91, 0.94)
**English language speakers (n = 303)**						
Brushing-SF	0.90 (0.87, 0.92)	0.84 (0.78, 0.88)	0.92 (0.89, 0.94)	0.90 (0.87, 0.92)	0.85 (0.79, 0.88)	0.92 (0.90, 0.94)
Flossing-SF	0.92 (0.90, 0.94)	0.87 (0.84, 0.90)	0.95 (0.93, 0.97)	0.93 (0.91, 0.94)	0.88 (0.85, 0.90)	0.95 (0.93, 0.97)
Dental Care-SF	0.93 (0.91, 0.94)	0.93 (0.91, 0.95)	0.93 (0.90, 0.95)	0.93 (0.91, 0.94)	0.93 (0.91, 0.95)	0.92 (0.90, 0.94)
**Sex**						
**Males (n = 105)**						
Brushing-SF	0.89 (0.84, 0.92)	0.88 (0.83, 0.91)	0.93 (0.90, 0.95)	0.89 (0.85, 0.92)	0.89 (0.84, 0.92)	0.93 (0.90, 0.95)
Flossing-SF	0.93 (0.90, 0.95)	0.88 (0.83, 0.91)	0.94 (0.91, 0.97)	0.93 (0.90, 0.95)	0.90 (0.85, 0.92)	0.94 (0.90, 0.97)
Dental Care-SF	0.91 (0.87, 0.93)	0.93 (0.89, 0.95)	0.92 (0.88, 0.95)	0.91 (0.88, 0.94)	0.93 (0.88, 0.95)	0.92 (0.87, 0.95)
**Females (n = 397)**						
Brushing-SF	0.91 (0.90, 0.92)	0.85 (0.82, 0.88)	0.94 (0.92, 0.95)	0.91 (0.90, 0.93)	0.86 (0.82, 0.88)	.938 (.925, .950)
Flossing-SF	0.93 (0.92, 0.94)	0.87 (0.84, 0.89)	0.96 (0.95, 0.97)	0.94 (0.93, 0.95)	0.87 (0.84, 0.89)	0.96 (0.95, 0.97)
Dental Care-SF	0.91 (0.90, 0.93)	0.92 (0.90, 0.94)	0.93 (0.91, 0.94)	0.91 (0.90, 0.93)	0.92 (0.90, 0.93)	0.93 (0.91, 0.94)
**Marital status**						
**Single (n = 314)**						
Brushing-SF	0.90 (0.88, 0.92)	0.86 (0.83, 0.89)	0.94 (0.92, 0.95)	0.90 (0.88, 0.92)	0.87 (0.83, 0.89)	0.94 (0.92, 0.95)
Flossing-SF	0.92 (0.91, 0.94)	0.87 (0.84, 0.89)	0.95 (0.94, 0.96)	0.93 (0.91, 0.94)	0.88 (0.85, 0.90)	0.96 (0.94, 0.97)
Dental Care-SF	0.91 (0.89, 0.92)	0.92 (0.90, 0.94)	0.93 (0.91, 0.94)	0.91 (0.89, 0.92)	0.92 (0.90, 0.94)	0.93 (0.91, 0.94)
**Married (n = 188)**						
Brushing-SF	0.92 (0.89, 0.93)	0.85 (0.81, 0.88)	0.93 (0.90, 0.95)	0.92 (0.90, 0.94)	0.86 (0.81, 0.89)	0.93 (0.91, 0.95)
Flossing-SF	0.94 (0.92, 0.92)	0.87 (0.84, 0.90)	0.96 (0.94, 0.97)	0.94 (0.93, 0.95)	0.88 (0.84, 0.90)	0.96 (0.94, 0.97)
Dental Care-SF	0.92 (0.90, 0.94)	0.93 (0.90, 0.95)	0.92 (0.89, 0.94)	0.92 (0.90, 0.94)	0.93 (0.90, 0.95)	0.92 (0.89, 0.94)

**Table 13.** Descriptives for general social support scales in the full sample, and by language preference, sex, and marital status

		LANGUAGE		SEX		MARITAL STATUS	
	Full Sample N=502 Mean (SD)	English n=303 mean (SD)	Spanish n=199 mean (SD)	Males n=105 mean (SD)	Females n=397 mean (SD)	Single n=314 mean (SD)	Partnered/Married n=188 mean (SD)
Interpersonal Support Evaluation List (ISEL-12)^2, 3^	24.69 (7.35)	24.56 (7.69)	24.88 (6.82)	23.30 (7.51)	25.06 (7.27)	23.69 (7.49)	26.36 (6.81)
ISEL-appraisal^2, 3^	8.72 (2.67)	8.70 (2.79)	8.74 (2.50)	8.13 (2.78)	8.87 (2.62)	8.38 (2.74)	9.29 (2.45)
ISEL-belonging^3^	7.91 (2.89)	7.74 (3.05)	8.17 (2.60)	7.48 (2.81)	8.03 (2.90)	7.56 (2.96)	8.50 (2.67)
ISEL-tangible^3^	8.06 (2.76)	8.11 (2.85)	7.97 (2.62)	7.69 (2.87)	8.16 (2.73)	7.75 (2.79)	8.56 (2.63)
Multidimensional Scale of Perceived Social Support (MSPSS)^2^	5.79 (1.66)	5.84 (1.63)	5.70 (1.71)	5.35 (1.88)	5.91 (1.58)	5.68 (1.65)	5.97 (1.67)
MSPSS-Family^2, 3^	5.33 (1.75)	5.32 (1.76)	5.34 (1.75)	4.99 (1.83)	5.42 (1.73)	5.12 (1.77)	5.67 (1.67)
MSPSS-Friends^2^	5.06 (1.76)	5.14 (1.79)	4.93 (1.72)	4.72 (1.95)	5.15 (1.70)	5.08 (1.73)	5.02 (1.82)
MSPSS-Significant Other^2, 3^	5.65 (1.72)	5.74 (1.69)	5.51 (1.75)	5.13 (1.96)	5.78 (1.62)	5.49 (1.73)	5.90 (1.67)
Modified Medical Outcomes Study Social Support Survey (mMOS)^2, 3^	3.89 (1.05)	3.86 (1.09)	3.94 (0.98)	3.70 (1.18)	3.95 (1.02)	3.76 (1.13)	4.14 (0.88)
mMOS-emotional	3.62 (1.00)	3.70 (1.01)	3.49 (0.99)	3.28 (1.12)	3.78 (0.92)	3.55 (0.91)	3.73 (1.15)
mMOS-tangible	3.71 (0.98)	3.76 (0.94)	3.63 (1.08)	3.38 (1.11)	3.87 (0.90)	3.63 (0.85)	3.83 (1.18)

1 Significant differences by language at p<0.05 level;

2 Significant differences by sex at p<0.05 level;

3 Significant differences by marital status at p<0.05 level

**Table 14.** Spearman correlation coefficients for assessing OHBSS-SF scales’ convergent validity with general social support scales.

OHBSS-SF	ISEL_all	ISEL_app	ISEL_belong	ISEL_tangible	MSPSS_Tot	MSPSS_sig	MSPSS_Fam	MSPSSFr	mMOS	mMOStang	mMOSemot
Full Sample N=502											
BF-SF	0.18**	0.15**	0.17**	0.17**	0.18**	0.13**	0.23**	0.11*	0.22**	0.21**	0.21**
BP-SF	0.14**	0.13**	0.10	0.13**	0.13**	0.09	0.13**	0.13**	0.15**	0.14**	0.14**
BO-SF	0.02	−0.03	0.07	0.01	0.10*	0.03	0.07	0.15**	0.09*	0.09*	0.08
FF-SF	0.17**	.13**	0.16**	0.15**	0.18**	0.13**	0.23**	0.12**	0.21**	0.21**	0.19**
FP-SF	0.19**	.17**	0.14**	0.19**	0.17**	0.09*	0.17**	0.18**	0.19**	0.18**	0.17**
FO-SF	0.03	−0.03	0.09*	0.02	0.10*	0.02	0.06	0.15**	0.09	0.09	0.07
DF-SF	0.25**	0.22**	0.22**	0.21**	0.23**	0.16**	0.29**	0.17**	0.27**	0.27**	0.26**
DP-SF	0.21**	0.21**	0.16**	0.18**	0.17**	0.10*	0.15**	0.18**	0.24**	0.24**	0.24**
DO-SF	0.09*	0.06	0.14**	0.05	0.15**	0.05	0.11*	0.19**	0.14**	0.13**	0.13**
English language n=303											
	ISEL_all	ISEL_app	ISEL_belong	ISEL_tangible	MSPSS_Tot	MSPSS_sig	MSPSS_Fam	MSPSSFr	mMOS	mMOStang	mMOSemot
BF-SF	0.20**	0.14*	0.19**	0.20**	0.21**	0.16**	0.28**	0.12*	0.20**	0.21**	0.18**
BP-SF	0.12*	0.09	0.09	0.14*	0.12*	0.06	0.11	0.14*	0.10	0.09	0.08
BO-SF	0.09	0.02	0.14*	0.07	0.13*	0.05	0.08	0.20**	0.09	0.11	0.06
FF-SF	0.17**	0.11	0.19**	0.16**	0.21**	0.16**	0.26**	0.13*	0.19**	0.20**	0.16**
FP-SF	0.19**	0.15*	0.15**	0.20**	0.15**	0.05	0.12*	0.20**	0.14*	0.13*	0.12*
FO-SF	0.10	0.01	0.17**	0.06	0.12*	0.04	0.09	0.18**	0.08	0.09	0.05
DF-SF	0.23**	0.19**	0.20**	0.20**	0.27**	0.20**	0.33**	0.19**	0.25**	0.26**	0.22**
DP-SF	0.17**	0.10**	0.12*	0.17**	0.15**	0.08	0.11	0.20**	0.18**	0.19**	0.17**
DO-SF	0.14*	0.08	0.18**	0.10	0.18**	0.09	0.14*	0.23**	0.13*	0.14*	0.10
Spanish language n=199											
	ISEL_all	ISEL_app	ISEL_belong	ISEL_tangible	MSPSS_Tot	MSPSS_sig	MSPSS_Fam	MSPSSFr	mMOS	mMOStang	mMOSemot
BF-SF	0.15*	.163*	.110	0.136	.142*	.094	.165*	.122	.236**	.202**	.258**
BP-SF	0.16*	.202**	.097	0.132	.165*	.147*	.176*	.122	.239**	.220**	.244**
BO-SF	−0.10	−.121	−.071	−0.081	.045	−0.013	.040	.076	.087	.053	.115
FF-SF	0.16*	.159*	.105	.152*	.146*	.091	.176*	.126	.233**	.218**	.229**
FP-SF	0.19**	.200**	.128	.177*	.194**	.149*	.233**	.150*	.280**	.270**	.269**
FO-SF	−0.09	−.102	−.064	−.065	.049	−.017	.030	.094	.092	.075	.102
DF-SF	0.29**	.269**	.250**	.239**	.185**	.115	.226**	.154*	.311**	.284**	.320**
DP-SF	0.27**	.289**	.223**	0.21**	0.20**	.154*	.211**	.166*	.344**	.328**	.345**
DO-SF	0.01	.004	.060	−.035	.097	.004	.073	.133	.144*	.092	.192**
s											
	ISEL_all	ISEL_app	ISEL_belong	ISEL_tangible	MSPSS_Tot	MSPSS_sig	MSPSS_Fam	MSPSSFr	mMOS	mMOStang	mMOSemot
BF-SF	0.24*	0.19	0.28**	0.16	0.10	0.12	0.18	0.02	0.22*	0.21*	0.22*
BP-SF	0.17	0.09	0.17	0.21*	0.13	0.12	0.07	0.16	0.16	0.16	0.14
BO-SF	0.01	−0.07	0.07	0.03	−0.02	−0.03	−0.10	0.07	0.06	0.05	0.05
FF-SF	0.28**	0.26**	0.31**	0.18	0.17	0.18	0.24*	0.06	0.29**	0.29**	0.27**
FP-SF	0.23*	0.14	0.22*	0.25**	0.12	0.07	0.08	0.15	0.20*	0.20*	0.18
FO-SF	0.01	−0.07	0.09	−0.004	0.04	0.03	0.01	0.07	0.08	0.06	0.07
DF-SF	0.29**	0.29**	0.27**	0.21*	0.14	0.13	0.23*	0.04	0.30**	0.30**	0.30**
DP-SF	0.22*	0.16	0.21*	0.22*	0.10	0.08	0.03	0.14	0.18	0.18	0.17
DO-SF	0.01	−0.04	0.05	0.01	0.00	−0.01	−0.02	0.03	0.10	0.09	0.09
Females n = 397											
	ISEL_all	ISEL_app	ISEL_belong	ISEL_tangible	MSPSS_Tot	MSPSS_sig	MSPSS_Fam	MSPSSFr	mMOS	mMOStang	mMOSemot
BF-SF	0.17**	0.13**	0.14**	0.17**	0.19**	0.12*	0.24**	0.14**	0.22**	0.20**	0.21**
BP-SF	0.11*	0.13**	0.07	0.10*	0.12*	0.06	0.14**	0.11*	0.13**	0.12*	0.12*
BO-SF	0.03	−0.02	0.07	0.01	0.14**	0.05	0.11*	0.18**	0.10*	0.10*	0.09
FF-SF	0.13**	0.09	0.12*	0.14**	0.17**	0.10*	0.22**	0.14**	0.18**	0.18**	0.16**
FP-SF	0.16**	0.16**	0.11*	0.17**	0.16**	0.07	0.18**	0.18**	0.17**	0.17**	0.15**
FO-SF	0.03	−0.02	0.09	0.02	0.11*	0.01	0.07	0.17**	0.08	0.09	0.06
DF-SF	0.24**	0.21**	0.21**	0.22**	0.26**	0.17**	0.31**	0.21**	0.27**	0.26**	0.26**
DP-SF	0.19**	0.21**	0.14**	0.16**	0.17**	0.09	0.17**	0.19**	0.25**	0.25**	0.25**
DO-SF	0.11*	0.07	0.15**	0.06	0.18**	0.06	0.14**	0.23**	0.14**	0.13**	0.14**
Single n = 314											
	ISEL_all	ISEL_app	ISEL_belong	ISEL_tangible	MSPSS_Tot	MSPSS_sig	MSPSS_Fam	MSPSSFr	mMOS	mMOStang	mMOSemot
BF-SF	0.20**	0.15*	0.18**	0.19**	0.24**	0.20**	0.31**	0.14*	0.25**	0.24**	0.23**
BP-SF	0.14*	0.13*	0.11	0.14*	0.15**	0.11	0.15**	0.14*	0.16**	0.16**	0.14*
BO-SF	0.05	0.004	0.11	0.02	0.13*	0.07	0.10	0.17**	0.13*	0.12*	0.12*
FF-SF	0.16**	0.11*	0.16**	0.15**	0.24**	0.19**	0.29**	0.14*	0.22**	0.23**	0.19**
FP-SF	0.21**	0.18**	0.16**	0.22**	0.18**	0.09	0.18**	0.20**	0.20**	0.20**	0.17**
FO-SF	0.04	−0.009	0.11*	−0.003	0.13*	0.06	0.11	0.15**	0.17*	0.11	0.10
DF-SF	0.22**	0.20**	0.20**	0.20**	0.29**	0.21**	0.36**	0.20**	0.27**	0.27**	0.25**
DP-SF	0.20**	0.22**	0.16**	0.16**	0.18**	0.12*	0.16**	0.19**	0.23**	0.23**	0.23**
DO-SF	0.07	0.04	0.11*	0.03	0.18**	0.11	0.17**	0.17**	0.15**	0.14*	0.14*
Partnered/Married n = 188											
	ISEL_all	ISEL_app	ISEL_belong	ISEL_tangible	MSPSS_Tot	MSPSS_sig	MSPSS_Fam	MSPSSFr	mMOS	mMOStang	mMOSemot
BF-SF	0.14	0.13	0.13	0.12	0.06	−0.01	0.08	0.08	0.15*	0.13	0.16*
BP-SF	0.11	0.12	0.07	0.11	0.10	0.05	0.09	0.12	0.10	0.07	0.12
BO-SF	0.001	−0.06	0.03	0.03	0.05	−0.03	0.02	0.13	0.04	0.05	0.02
FF-SF	0.15*	0.12	0.14	0.12	0.07	−0.01	0.09	0.10	0.15*	0.14	0.15*
FP-SF	0.13	0.11	0.10	0.14	0.14	0.07	0.13	0.16*	0.14	0.12	0.16*
FO-SF	0.05	−0.04	0.09	0.08	0.06	−0.04	0.02	0.16*	0.06	0.07	0.03
DF-SF	0.27**	0.25**	0.25**	0.22**	0.13	0.06	0.16*	0.13	0.27**	0.25**	0.27**
DP-SF	0.21**	0.18*	0.16*	0.21**	0.13	0.05	0.11	0.17*	0.26**	0.25**	0.25**
DO-SF	0.17*	0.12	0.22**	0.13	0.10	−0.03	0.03	0.23**	0.15*	0.13	0.15*

** Correlation is significant at the 0.01 level (2-tailed).

* Correlation is significant at the 0.05 level (2-tailed).

Heatmap guide: Significant correlations ≥ 0.10 shaded light blue, correlations ≥ 0.20 shaded dark blue (darker color shading for stronger correlation).

*OHBSS-SF:* Oral health behavior social support short form scales (there are nine scales, comprised of 17 required items, reflecting behavior-source of support group combinations). Behaviors: B=Brushing, F=Flossing, D=Dental Care; Source of support: F=Family, P=Providers, O=Others/Friends.

*ISEL-12:* Interpersonal Support Evaluation List; app=appraisal subscale, belong=belonging subscale, tangible=tangible subscale.

*MSPSS:* Multidimensional Scale of Perceived Social Support; Sig=significant other subscale, Fam=family subscale, Fr=friend subscale

*mMOS:* Modified Medical Outcomes Study-Social Support Survey; tang=tangible subscale, emot=emotional subscale

**Table 15.** Descriptives for the SASH and MDAS scales in the full sample, and by language preference, sex, and marital status

		LANGUAGE		SEX		MARITAL STATUS	
	Full Sample N=502 Mean (SD)	English n=303 mean (SD)	Spanish n=199 mean (SD)	Males n=105 mean (SD)	Females n=397 mean (SD)	Single n=314 mean (SD)	Married n=188 mean (SD)
SASH^1,3^	2.55 (0.87)	3.08 (0.60)	1.73 (0.49)	2.57 (0.89)	2.54 (0.86)	2.77 (0.83)	2.18 (0.80)
SASH_lang^1,3^	2.66 (1.21)	3.45 (0.83)	1.47 (0.56)	2.67 (1.22)	2.66 (1.21)	2.98 (1.16)	2.13 (1.11)
SASH_SOC^1,3^	2.26 (0.68)	2.43 (0.66)	2.01 (0.62)	2.32 (0.71)	2.25 (0.67)	2.34 (0.70)	2.13 (0.62)
MDAS^1,3^	13.06 (6.03)	13.77 (6.13)	11.98 (5.71)	13.10 5.90	13.05 (6.07)	13.90 (6.05)	11.64 (5.74)

1 Significant differences by language at p<0.05 level;

2 Significant differences by sex at p<0.05 level;

3 Significant differences by marital status at p<0.05 level.

SASH = Short Acculturation Scale for Hispanics; Lang = language subscale, SOC=social interactions subscale.

MDAS=Modified Dental Anxiety Scale

**Table 16.** Spearman correlation coefficients for assessing OHBSS-SF scales’ divergent validity with SASH and MDAS, in the full sample and by language preference, sex and marital status

Full sample (N=502)				
OHBSS-SF	SASH	SASH_Lang	SASH_SOC	MDAS
BF-SF	−0.13**	−0.15**	−0.03	−0.06
BP-SF	−0.02	−0.03	0.02	−0.07
BO-SF	−0.01	−0.02	0.02	0.02
FF-SF	−0.09*	−0.11*	−0.003	−0.02
FP-SF	0.01	−0.003	0.03	−0.06
FO-SF	0.01	0.01	0.03	0.02
DF-SF	−0.06	−0.08	0.02	−0.06
DP-SF	−0.04	−0.06	0.03	−0.10*
DO-SF	0.01	−0.01	0.05	0.014
English (n=303)				
OHBSS-SF	SASH	SASH_Lang	SASH_SOC	MDAS
BF-SF	−0.11	−0.13*	−0.01	−0.10
BP-SF	−0.02	−0.02	0.01	−0.11
BO-SF	−0.02	−0.02	0.02	−0.04
FF-SF	−0.06	−0.07	0.03	−0.03
FP-SF	0.02	0.01	0.05	−0.12*
FO-SF	−0.02	−0.02	0.02	−0.03
DF-SF	−0.03	−0.05	0.02	−0.07
DP-SF	0.02	−0.01	0.08	−0.13*
DO-SF	0.00	0.01	0.01	−0.001
Spanish (n=199)				
OHBSS-SF	SASH	SASH_Lang	SASH_SOC	MDAS
BF-SF	0.09	0.09	0.04	0.07
BP-SF	0.14*	0.13	0.10	0.04
BO-SF	0.04	0.02	0.05	0.12
FF-SF	0.05	0.03	0.04	0.03
FP-SF	0.10	0.12	0.03	0.06
FO-SF	0.06	0.04	0.03	0.10
DF-SF	0.05	0.02	0.08	0.01
DP-SF	0.08	0.08	0.04	0.01
DO-SF	0.10	0.03	0.13	0.05
Males (n = 105)				
OHBSS-SF	SASH	SASH_Lang	SASH_SOC	MDAS
BF-SF	−0.17	−0.15	−0.15	−0.24*
BP-SF	−0.01	0.04	−0.10	−0.21*
BO-SF	0.12	0.18	0.02	−0.11
FF-SF	−0.14	−0.16	−0.06	−0.26*
FP-SF	0.02	0.06	−0.08	−0.22*
FO-SF	0.17	0.15	0.11	−0.16
DF-SF	−0.11	−0.11	−0.10	−0.16
DP-SF	−0.02	0.02	−0.11	−0.23*
DO-SF	0.10	0.10	0.02	−0.01
Females (n = 397)				
OHBSS-SF	SASH	SASH_Lang	SASH_SOC	MDAS
BF-SF	−0.12*	−0.15**	−0.001	−0.02
BP-SF	−0.03	−0.06	0.06	−0.03
BO-SF	−0.05	−0.06	0.02	0.05
FF-SF	−0.08	−0.10	0.02	0.03
FP-SF	0.01	−0.02	0.07	−0.02
FO-SF	−0.02	−0.02	0.01	0.05
DF-SF	−0.05	−0.07	0.05	−0.03
DP-SF	−0.05	−0.09	0.08	−0.06
DO-SF	−0.01	−0.03	0.06	0.02
Single (n = 314)				
OHBSS-SF	SASH	SASH_Lang	SASH_SOC	MDAS
BF-SF	−0.15**	−0.16**	−0.03	−0.04
BP-SF	−0.02	−0.03	0.03	−0.10
BO-SF	−0.01	−0.03	0.05	0.05
FF-SF	−0.05	−0.07	0.03	0.05
FP-SF	0.04	0.04	0.07	−0.06
FO-SF	0.02	−0.001	0.07	0.06
DF-SF	−0.05	−0.07	0.01	−0.05
DP-SF	−0.03	−0.05	0.05	−0.11*
DO-SF	−0.001	−0.01	0.04	0.06
Married (n = 188)				
OHBSS-SF	SASH	SASH_Lang	SASH_SOC	MDAS
BF-SF	−0.059	−0.069	−0.01	−0.06
BP-SF	0.000	−0.016	0.02	0.001
BO-SF	−0.081	−0.070	−0.07	−0.07
FF-SF	−0.086	−0.096	−0.03	−0.09
FP-SF	−0.016	−0.022	−0.02	−0.04
FO-SF	−0.055	−0.036	−0.10	−0.09
DF-SF	−0.035	−0.063	0.05	−0.04
DP-SF	−0.037	−0.060	0.02	−0.03
DO-SF	−0.021	−0.048	0.04	−0.10

Note.

** Correlation is significant at the 0.01 level (2-tailed).

* Correlation is significant at the 0.05 level (2-tailed). SASH = Short Acculturation Scale for Hispanics; Lang = language subscale, SOC=social interactions subscale. MDAS=Modified Dental Anxiety Scale

**Table 17.** Descriptives for oral health behaviors and status in the full sample, and by language preference, sex, and marital status

		LANGUAGE		SEX		MARITAL STATUS	
	Full Sample N=502	English n=303	Spanish n=199	Males n=105	Females n=397	Single n=314	Married n=188
Brushing, past week frequency, mean (SD)	12.2 (6.8)	11.9 (5.6)	12.6 (8.0)	11.1 (5.9)	12.5 (6.8)	11.8 (6.1)	12.8 (7.5)
Flossing, past week frequency, mean (SD)^2^	5.9 (6.2)	5.6 (5.3)	6.4 (7.3)	4.7 (4.9)	6.2 (6.5)	5.6 (5.5)	6.3 (7.2)
Dental visit in the last year^2^	266 (53%)	172 (57%)	94 (47%)	41 (39%)	225 (57%)	169 (54%)	97 (52%)
Fair/poor self-rated oral health	266 (53%)	156 (51%)	110 (56%)	58 (55%)	208 (52%)	171 (54%)	95 (51%)
Self-reported periodontal disease	125 (25%)	76 (25%)	49 (25%)	29 (28%)	96 (24%)	73 (23%)	52 (28%)
Missing 1+ teeth due to disease	184 (37%)	91 (31%)	93 (47%)	40 (38%)	144 (36%)	108 (34%)	76 (40%)

1 Significant differences by language at p≤0.05 level;

2 Significant differences by sex at p≤0.05 level;

3 Significant differences by marital status at p≤0.05 level.

**Table 18.** Spearman Correlations between the OHBSS-SF and brushing and flossing, in the full sample, and by language preference, sex, and marital status

BRUSHING FREQUENCY (# in past week)							
		LANGUAGE		SEX		MARITAL STATUS	
OHBSS-SF	Full Sample N=502	English n=303	Spanish n=199	Males n=105	Females n=397	Single n=314	Married n=188
BF-SF	0.07	0.09	0.06	0.33**	0.02	0.11	0.02
BP-SF	0.09*	0.08	0.10	0.09	0.08	0.10	0.07
BO-SF	0.04	0.01	0.06	0.18	0.01	0.04	0.04
FF-SF	0.14**	0.07	0.22**	0.31**	0.10*	0.10	0.18*
FP-SF	0.11*	0.07	0.15*	0.10	0.10	0.12*	0.09
FO-SF	0.04	0.04	0.05	0.15	0.02	0.04	0.05
FLOSSING FREQUENCY (# in past week)							
	Full Sample N=502	English n=303	Spanish n=199	Males n=105	Females n=397	Single n=314	Married n=188
BF-SF	0.09*	0.10	0.07	0.19*	0.07	0.09	0.08
BP-SF	0.14**	0.09	0.20**	0.25**	0.10*	0.08	0.21**
BO-SF	0.10*	0.07	0.14*	0.001	0.13*	0.19*	0.10
FF-SF	0.23**	0.14*	0.32**	0.25**	0.22**	0.17**	0.28**
FP-SF	0.19**	0.11	0.28**	0.28**	0.15**	0.15**	0.23**
FO-SF	0.20**	0.16**	0.26**	0.14	0.20**	0.22**	0.19*

** Correlation is significant at the 0.01 level (2-tailed).

* Correlation is significant at the 0.05 level (2-tailed)

**TABLE 19.** OHBSS-SF dental care social support scales by dental visit in the last year, in the full sample, and by language preference, sex, and marital status

	Dental Visit in Last Year mean±SD	No Dental Visit in Last Year mean±SD
**OHBSS-SF, Full Sample (N=502)**	**n=266**	**n=236**
DF-SF	2.20±1.24	2.18±1.23
DP-SF*	3.19±0.94	2.77±1.26
DO-SF	0.99±1.18	1.01±1.11
**English (n=303)**	**n=172**	**n=131**
DF-SF	2.15±1.22	2.05±1.24
DP-SF*	3.18±0.92	2.57±1.30
DO-SF	0.97±1.22	0.99±1.12
**Spanish (n=199)**	**n=94**	**n=105**
DF-SF	2.29±1.28	2.34±1.19
DP-SF	3.23±0.97	3.02±1.16
DO-SF	1.00±1.11	1.04±1.09
**Males (n=105)**	**n=41**	**n=64**
DF-SF	2.28±1.16	2.13±1.17
DP-SF	3.01±1.03	2.62±1.26
DO-SF	0.93±1.18	0.79±0.88
**Females (n=397)**	**n=225**	**n=172**
DF-SF	2.19±1.26	2.21±1.25
DP-SF*	3.23±0.92	2.82±1.25
DO-SF	1.00±1.18	1.10±1.17
**Single (n=314)**	**n=169**	**n=145**
DF-SF	2.19±1.21	2.07±1.23
DP-SF*	3.24±0.91	2.64±1.26
DO-SF	1.06±1.24	1.02±1.12
**Married (n=188)**	**n=97**	**n=91**
DF-SF	2.22±1.30	2.36±1.20
DP-SF	3.13±0.97	2.97±1.23
DO-SF	0.86±1.07	1.00±1.09

* significant difference in dental care social support scale short-form scores, p≤0.05;

D=dental care, F=Family, P=Providers, O=Others/Friends, SF=Short-Form

**TABLE 20.** OHBSS-SF dental care social support scales by self-reported periodontal disease status, in the full sample, and by language preference, sex, and marital status

	No Periodontal Disease mean±SD	Periodontal Disease mean±SD
**OHBSS-SF, Full Sample (N=502)**	**n=377**	**n=125**
DF-SF	2.24±1.24	2.05±1.22
DP-SF*	3.06±1.07	2.80±1.24
DO-SF	1.01±1.17	0.94±1.06
**English (n=303)**	**n=227**	**n=76**
DF-SF	2.15±1.23	2.01±1.22
DP-SF	2.98±1.11	2.73±1.20
DO-SF	0.98±1.18	1.00±1.18
**Spanish (n=199)**	**n=150**	**n=49**
DF-SF	2.38±1.23	2.11±1.24
DP-SF	3.19±0.98	2.90±1.31
DO-SF	1.08±1.16	0.85±0.86
**Males (n=105)**	**n=76**	**n=29**
DF-SF	2.27±1.12	1.98±1.29
DP-SF	2.89±1.15	2.48±1.26
DO-SF	0.78±0.99	1.02±1.02
**Females (n=397)**	**n=301**	**n=96**
DF-SF	2.24±1.27	2.07±1.20
DP-SF	3.11±1.04	2.89±1.23
DO-SF	1.08±1.21	0.92±1.08
**Single (n=314)**	**n=241**	**n=73**
DF-SF	2.19±1.22	1.96±1.23
DP-SF	3.01±1.11	2.81±1.19
DO-SF	1.05±1.20	1.15±1.15
**Married (n=188)**	**n=136**	**n=52**
DF-SF	2.33±1.27	2.17±1.21
DP-SF	3.16±0.99	2.79±1.33
DO-SF	0.96±1.13	0.85±0.94

* significant difference in dental care social support scale short-form scores, p≤0.05;

D=dental care, F=Family, P=Providers, O=Others/Friends, SF=Short-Form

**TABLE 21.** OHBSS-SF dental care social support scales by self-rated oral health status, in the full sample, and by language preference, sex, and marital status

	Excellent/Very Good/Good mean±SD	Fair/Poor mean±SD
**OHBSS-SF, Full Sample (N=502)**	**n=236**	**n=266**
DF-SF*	2.46±1.14	1.96±1.26
DP-SF*	3.18±0.95	2.83±1.23
DO-SF*	1.19±1.22	0.82±1.05
**English (n=303)**	**n=147**	**n=156**
DF-SF*	2.36±1.14	1.88±1.27
DP-SF*	3.19±0.90	2.65±1.27
DO-SF*	1.13±1.22	0.84±1.13
**Spanish (n=199)**	**n=89**	**n=110**
DF-SF*	2.64±1.13	2.06±1.25
DP-SF	3.16±1.03	3.09±1.12
DO-SF*	1.30±1.22	0.81±0.94
**Males (n=105)**	**n=47**	**n=58**
DF-SF*	2.44±0.97	1.98±1.27
DP-SF	2.98±0.94	2.61±1.34
DO-SF	0.91±1.08	0.79±0.93
**Females (n=397)**	**n=189**	**n=208**
DF-SF*	2.47±1.18	1.95±1.26
DP-SF*	3.23±0.95	2.90±1.19
DO-SF*	1.15±1.25	0.84±1.08
**Single (n=314)**	**n=143**	**n=171**
DF-SF*	2.44±1.15	1.89±1.23
DP-SF*	3.17±0.94	2.80±1.24
DO-SF*	1.25±1.27	0.87±1.08
**Married (n=188)**	**n=93**	**n=95**
DF-SF*	2.50±1.14	2.08±1.32
DP-SF	3.20±0.96	2.91±1.21
DO-SF*	1.11±1.13	0.75±1.00

* significant difference in dental care social support scale short-form scores, p≤0.05;

D=dental care, F=Family, P=Providers, O=Others/Friends, SF=Short-Form

**TABLE 22.** OHBSS-SF dental care social support scales by self-reported any missing teeth due to disease, in the full sample, and by language preference, sex, and marital status

	Not Missing Any Teeth mean±SD	Missing Teeth mean±SD
**OHBSS-SF, Full Sample (N=502)**	**n=318**	**n=184**
DF-SF	2.16±1.24	2.25±1.22
DP-SF	2.95±1.17	3.07±1.01
DO-SF	0.98±1.14	1.04±1.16
**English (n=303)**	**n=212**	**n=91**
DF-SF	2.06±1.20	2.23±1.29
DP-SF	2.91±1.16	2.94±1.10
DO-SF	0.91±1.12	1.14±1.30
**Spanish (n=199)**	**n=106**	**n=93**
DF-SF	2.36±1.30	2.27±1.16
DP-SF	3.05±1.20	3.20±0.91
DO-SF	1.11±1.18	0.93±0.99
**Males (n=105)**	**n=65**	**n=40**
DF-SF*	2.06±1.11	2.39±1.25
DP-SF	2.72±1.20	2.86±1.18
DO-SF	0.86±1.06	0.82±0.92
**Females (n=397)**	**n=253**	**n=144**
DF-SF	2.19±1.27	2.21±1.22
DP-SF	3.01±1.16	3.13±0.96
DO-SF	1.01±1.16	1.10±1.21
**Single (n=314)**	**n=206**	**n=108**
DF-SF	2.08±1.23	2.24±1.20
DP-SF	2.93±1.17	3.03±1.04
DO-SF	0.96±1.12	1.19±1.29
**Married (n=188)**	**n=112**	**n=76**
DF-SF	2.31±1.25	2.26±1.26
DP-SF	3.01±1.18	3.12±0.99
DO-SF	1.01±1.18	0.81±0.90

D=dental care, F=Family, P=Providers, O=Others/Friends, SF=Short-Form

**Table 23.** Descriptives for clinical oral health indicators in Subsample 1, and by language preference, sex, and marital status

		LANGUAGE		SEX		MARITAL STATUS	
	Subsample 1 n=41	English n=21	Spanish n=20	Males n=7	Females n=34	Single n=22	Married n=19
Decayed teeth – mean ± SD	2.0±2.3	2.69±3.5	1.2±2.0	2.6±2.0	1.5±2.4	2.1±2.8	1.2±1.7
Proportion of sites with bleeding on probing - mean ± SD	0.39±0.24	0.36±0.23	0.43±0.24	0.61±0.19	0.35±0.23	0.38±0.21	0.41±0.27

**Table 24.** Test-retest reliability of the OHBSS-SF in Subsample 2, and by language preference, sex, and marital status

Subsample 2 (N=56)	Family ICC (95% CI)	Provider ICC (95% CI)	Others/Friends ICC (95% CI)
Brushing	0.62* (0.43, 0.76)	0.68* (0.51, 0.80)	0.60* (0.41, 0.75)
Flossing	0.60 (0.40, 0.74)	0.67* (0.50, 0.80)	0.55 (0.33, 0.71)
Dental Care	0.69* (0.53, 0.81)	0.77** (0.64, 0.86)	0.66* (0.48, 0.78)
**LANGUAGE**			
**English (n =35)**			
Brushing	0.40 (0.08, 0.65)	0.55 (0.26, 0.74)	0.41 (0.10, 0.65)
Flossing	0.41 (0.10, 0.65)	0.53 (0.23, 0.73)	0.34 (0.01, 0.60)
Dental Care	0.47 (0.16, 0.69)	0.68* (0.45, 0.83)	0.57 (0.29, 0.76)
**Spanish (n = 21)**			
Brushing	0.85** (0.67, 0.94)	0.87** (0.69, 0.95)	0.83** (0.64, 0.93)
Flossing	0.80** (0.57, 0.91)	0.87** (0.71, 0.94)	0.85** (0.67, 0.94)
Dental Care	0.93** (0.84, 0.97)	0.92** (0.81, 0.97)	0.75* (0.47, 0.89)
**SEX**			
**Male (n =7)**			
Brushing	0.60 (−0.11, 0.92)	0.68* (0.02, 0.94)	0.65* (−0.10, 0.93)
Flossing	0.37 (−0.62, 0.86)	0.61* (−0.11, 0.92)	0.77** (0.17, 0.96)
Dental Care	0.79** (0.18, 0.96)	0.98** (0.88, 0.99)	0.89** (0.28, 0.98)
**Female (n = 49)**			
Brushing	0.63* (0.42, 0.77)	0.67* (0.49, 0.80)	0.60* (0.39, 0.75)
Flossing	0.64* (0.44, 0.78)	0.69*(0.51, 0.81)	0.52 (0.28, 0.70)
Dental Care	0.68* (0.50, 0.81)	0.71** (0.54, 0.82)	0.64* (0.45, 0.78)
**MARITAL STATUS**			
**Married (n =23)**			
Brushing	0.58 (0.23, 0.80)	0.72* (0.44, 0.87)	0.57 (0.22, 0.79)
Flossing	0.60* (0.27, 0.81)	0.67* (0.38, 0.85)	0.51 (0.13, 0.76)
Dental Care	0.81** (0.60, 0.91)	0.74* (0.47, 0.88)	0.50 (0.13, 0.75)
**Single (n = 33)**			
Brushing	0.66* (0.41, 0.81)	0.61* (0.34, 0.79)	0.59 (0.32, 0.77)
Flossing	0.60* (0.33, 0.78)	0.67* (0.42, 0.82)	0.52 (0.21, 0.73)
Dental Care	0.61* (0.34, 0.78)	0.80** (0.63, 0.90)	0.69* (0.46, 0.84)

Note.

* intra-class correlation coefficients (ICC) 0.60 to 0.74 = “good”;

** ICC ≥ 0.75 = “excellent”

## Data Availability

The datasets used and/or analyzed during the current study are available from the corresponding author on reasonable request.

## ABBREVIATIONS

AZ: Arizona
BF: Brushing social support from family
BO: Brushing social support from others/friends
BP: Brushing social support from health providers
CA: California
CI: Confidence interval
DF: Dental care social support from family
DO: Dental care social support from others/friends
DP: Dental care social support from health providers
DMFT: Decayed Missing Filled Teeth
FF: Flossing social support from family
FO: Flossing social support from others/friends
FP: Flossing social support from health providers
IRB: Institutional Review Board
ICC: Intra-class correlation
ISEL: Interpersonal Support Evaluation List-12 item social support scale
MDAS: Modified Dental Anxiety Scale
mMOS: Modified Medical Outcomes Study Social Support Survey
MSPSS: Multidimensional Scale of Perceived Social Support
NHANES: National Health and Nutrition Examination Survey
NIDCR: National Institutes of Dental and Craniofacial Research
OHBSS: Oral Health Behavior Social Support scales
OHBSS-SF: Oral Health Behavior Social Support Short-Form scales
SASH: Short Acculturation Scale for Hispanics
SDSU: San Diego State University
US: United States
